# Pressure Optimized PowEred Respirator (PROPER): A miniaturized wearable cleanroom and biosafety system for aerially transmitted viral infections such as COVID-19

**DOI:** 10.1016/j.ohx.2020.e00144

**Published:** 2020-10-06

**Authors:** Miracle Israel Nazarious, Thasshwin Mathanlal, Maria-Paz Zorzano, Javier Martin-Torres

**Affiliations:** a Group of Atmospheric Science, Department of Computer Science, Electrical and Space Engineering, Luleå, University of Technology, Luleå 97 187, Sweden; b Centro de Astrobiología (CSIC-INTA), Torrejon de Ardoz, 28850 Madrid, Spain; c School of Geosciences, University of Aberdeen, Meston Building, King's College, Aberdeen AB24 3UE, UK; d Instituto Andaluz de Ciencias de la Tierra (CSIC-UGR), 18100 Granada, Spain

**Keywords:** Do-It-Yourself, Personal Protective Equipment, PAPR, BSL-3, Overpressure, Cleanroom-aerosol-respiration-mask-biosafety

## Abstract

The supply of Personal Protective Equipment (PPE) in hospitals to keep the Health Care Professionals (HCP) safe taking care of patients may be limited, especially during the outbreak of a new disease. In particular, the face and body protective equipment is critical to prevent the wearer from exposure to pathogenic biological airborne particulates. This situation has been now observed worldwide during the onset of the COVID-19 pandemic. As concern over shortages of PPE at hospitals grows, we share with the public and makers’ community the Pressure Optimized PowEred Respirator (PROPER) equipment, made out of COTS components. It is functionally equivalent to a Powered Air Purifying Respirator (PAPR). PROPER, a hood-based system which uses open source and easily accessible components is low-cost, relatively passive in terms of energy consumption and mechanisms, and easy and fast to 3D print, build and assemble. We have adapted our experience on building clean room environments and qualifying the bioburden of space instruments to this solution, which is in essence a miniaturized, personal, wearable cleanroom. PROPER would be able to offer better protection than an N95 respirator mask, mainly because it is insensitive to seal fit and it shields the eyes as well. The PROPER SMS fabric is designed for single-use and not intended for reuse, as they may start to tear and fail but the rest of the parts can be disinfected and reused. We provide a set of guidelines to build a low-cost 3D printed solution for an effective PAPR system and describe the procedures to validate it to comply with the biosafety level 3 requirements. We have validated the prototype of PROPER unit for air flow, ISO class cleanliness level, oxygen and carbon-dioxide gas concentrations during exhalation, and present here these results for illustration. We demonstrate that the area inside the hood is more than 200 times cleaner than the external ambient without the operator and more than 175 times with the operator and in an aerosol exposed environment. We also include the procedure to clean and disinfect the equipment for reuse. PROPER may be a useful addition to provide protection to HCPs against the SARS-CoV-2 virus or other potential future viral diseases that are transmitted aerially.

**Specifications table**

**Table:** 

Hardware name	PRessure Optimized PowEred Respirator (PROPER)
Subject area	• Medical • Biosafety • Cleanroom • 3D printing
Hardware type	• Personal Protective Equipment (PPE) • Powered Air Purifying Respirator (PAPR) • Biosafety Level (BSL-3) laboratory equipment • Wearable cleanroom
Open Source License	GNU General Public License (GPL) 3.0
Cost of Hardware	185 GBP
Source File Repository	PROPER: Mendeley

## 1 Hardware in context

Personal Protective Equipment (PPE) is a crucial piece of everyday clothing for the healthcare personnel (HCP) to protect themselves from the exposure of potentially infectious patients and dangerous substances used in healthcare. However, during the onset of a pandemic, such as the one we are just experiencing worldwide with COVID-19, the supply of PPE in hospitals, clinics and healthcare centers, to keep the HCPs safe taking care of patients can be at times very limited [1]. Many hospitals are facing rapidly dwindling supplies of essential safety equipment such as respirators. This equipment is very important for the usual care of patients with COVID-19 as they prevent the wearer from exposure to pathogenic biological airborne particulates and from contact with contaminated surfaces. These airborne particulates or aerosols generated by the patients with COVID-19 pose additional risk to HCPs [2]. Patients in Intensive Care Unit (ICU) requiring respiratory support or life-saving procedures such as endotracheal intubation for mechanical ventilation and other procedures that cause a spray of aerosols carrying SARS-CoV-2 virus. These aerosols, unlike droplets, can suspend in the air for a longer duration and can be inhaled by the HCPs or others in the room since these particles are tiny enough to pass through surgical masks. Therefore, performing such life-saving procedures is hazardous to HCPs, caring for the patient and this requires improved levels of protection.

N95 respirators are widely used by HCPs against the aerosol spread of pathogens. However, since these masks must fit around the nose and mouth to make a perfect seal for effective filtration, a Powered Air Purifying Respirator (PAPR) system is the first choice for protection against aerosols and the production of these specialized respiratory hoods are in demand [3]. The hood benefits from no uncomfortable tight-fitting seals on or around the face, making it particularly suitable for racial/ethnic/religious groups that cannot shave facial hair or fail the FFP3 mask fit test. A fit test is not required for wearing a loose-fitting PAPR hood [4]. Hence, we decided to build PROPER, a simple, low-cost, fast and robust solution for a PAPR system called PRessure Optimized PowEred Respirator (PROPER).

As concern over shortages of PPE at hospitals worldwide grows, the Centers for Disease Control and Prevention (CDC) has provided recommendations for HCPs managing the COVID-19 outbreak to optimize the supply of PPE and equipment [5], [6]. The Food and Drug Administration (FDA) has recommended conservation strategies during this time period and issued Emergency Use Authorizations (EUAs) [7] to authorize respirators and other types of personal protective equipment, including all CDC’s National Institute for Occupational Safety and Health (NIOSH) approved particulate-filtering air purifying respirators (APRs) such as the facepiece respirators, elastomeric APRs, powered air purifying respirators, expired NIOSH-approved filtering facepiece respirators [2], [8], [9], and respirators that have been decontaminated pursuant to the terms and conditions of an authorized decontamination system [10]. In addition, non-NIOSH-approved disposable filtering facepiece respirators are permitted for use as well.

As the COVID-19 outbreak continues to expand globally, the supply chain for these devices will continue to be stressed if demand exceeds available supplies. The general public and the Makers’ community could be a vital resource to balance this widening difference in the demand and supply of PPE. There are numerous examples about the public and the Makers’ community working to produce equipment such as surgical masks, face shields, and gowns, extensively using 3D printing. There exists to date some other open source PAPR systems which are currently under development such as the PAPR by Imperial College London [4], OpenSourcePAPR [11], Open-Mask [12], Low-Cost PAPR [13], etc. These PAPR systems have similar functions as PROPER but their performance qualification has not been completed or stated clearly. With this paper, we aim to provide the public and the Makers’ community, a set of guidelines to build a low-cost 3D printed solution for an effective PAPR, reusable system and describe the procedures to validate it to comply with the biosafety level 3 requirements [14] and specific requirements by the World Health Organization (WHO) regarding decontamination and reusability during this COVID-19 outbreak [15].

Inspired by the 3 M™ Versaflo™ Powered Air Purifying Respirator (PAPR) system design [16], a static pressure air flow of 6–8.8 cubic feet per minute (CFM) equivalent to 170–250 L per minute (LPM), that is purified with a HEPA H14 corrugated filter (up to 0.3 µm particles with at least 99.97% efficiency) is placed inside the hood of the operator. HEPA H14 filter exhibits excellent performance in terms of filtration of pathogens such as bacteria and viruses, better than the clinically widely used N95, FFP2 or FFP3 filtering masks [4]. Compared to its commercial equivalent PAPR systems which is in high demand because of the COVID-19 pandemic, the PROPER which uses open source and easily accessible components is low-cost, relatively passive in terms of energy consumption and mechanisms, and easy and fast to 3D print and build the assembly. We adapted our knowledge and experience from building a cleanroom for qualifying the bioburden of space instruments [17] to design a miniaturized wearable cleanroom. With this approach, it is feasible to quantify the cleanliness level inside the hood and ensure utmost safety of the HCPs.

## 2 Hardware description

**Fig. 1:**
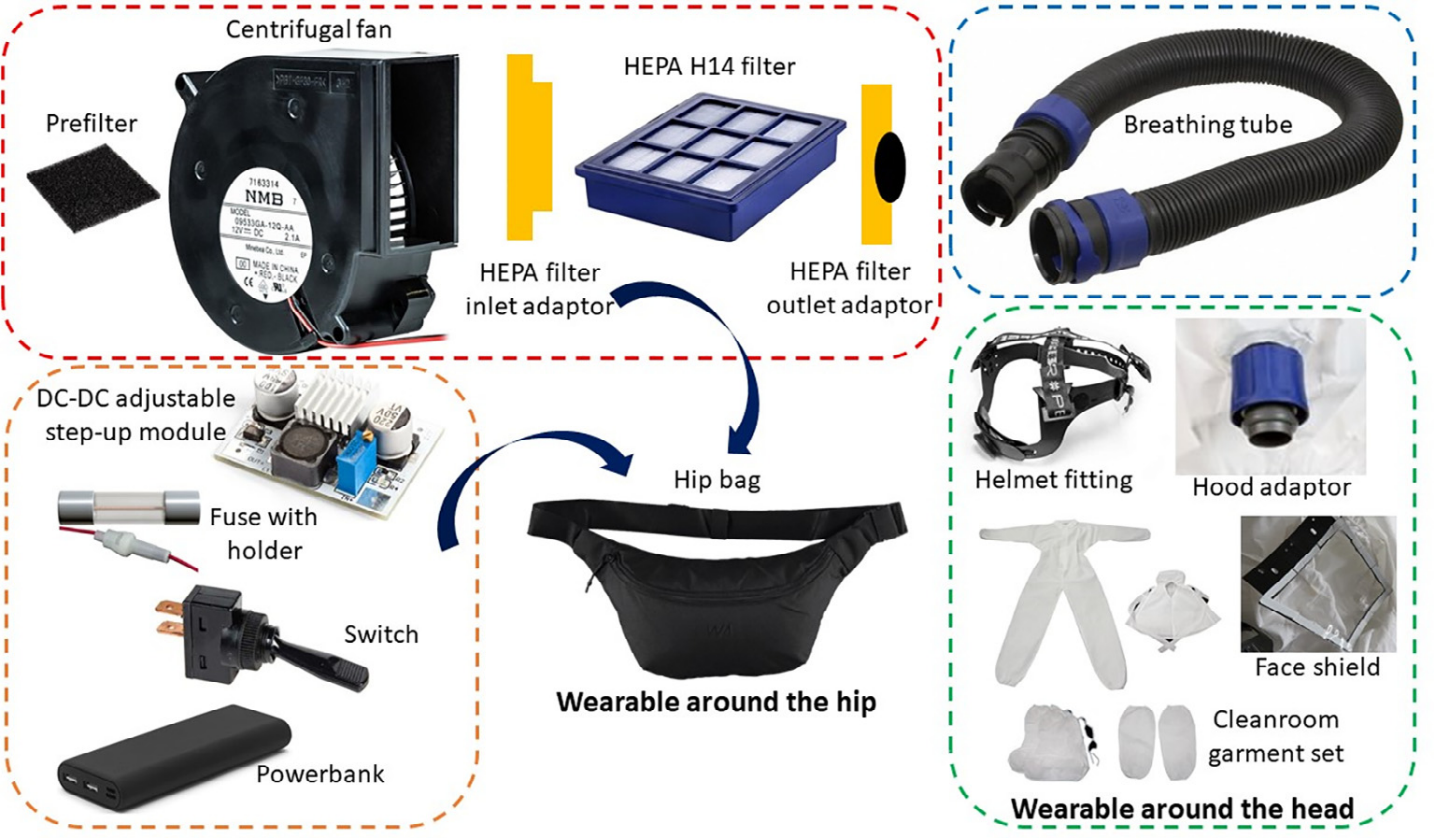
Schematic of the components used in the PROPER assembly.

PROPER assembly is comprised by four primary units, see Fig. 1: i) Hood, ii) Fan Filter Unit (FFU), iii) Breathing tube, and iv) Power supply and conditioning unit. PROPER is a three-piece equipment, a hood with a helmet fitting attached to the transparent face shield, a hip bag consisting of a centrifugal fan, HEPA H14 corrugated filter and the power supply and conditioning unit, and a breathing tube connecting the hood and the hip bag through adaptors. An overall ISO5 certified cleanroom garment with hood is normally worn as a PPE by HCPs. PROPER upgrades the existing hoods to a PAPR system providing additional protection from COVID-19 without demanding separate components to build the hood assembly. The components of Fig. 1, and the design criteria, are presented in the following subsections.

### 2.1 Hood

The hood is made of a Tyvek waterproof fabric and donned around the operator’s head with the neck and shoulder covered by the shroud. The hood of PROPER resembles a 3 M™ Versaflo™ S-433/533 hood with fabric that extends down over the chest making it better for protection from skin contamination [16]. In PROPER, the open cutting in the hood in front of the face is fitted with a 3D printed curved face shield frame and a transparent sheet (OHP film cut into the shape of the curved face shield frame). This screen provides a perfect sealing to block any back flow of air from outside to inside of the hood while creating a closed clean environment. The hood is made of Spunbond Meltblown Spunbond (SMS) Polypropylene fabric which is very breathable and hence, even under perfect closure, the exhaled air is partially “leaked” through the hood fabric and other tiny orifices in single flow direction (from overpressure inside to outside). Thus, the level of protection afforded by PROPER is almost always limited by the capacity of the seals to exclude intrusion of air from exterior to interior of the hood. The SMS fabric also has additional protective properties such as a greater level of chemical protection, a limited degree of flame spread resistance, and finally it also helps to reduce the buildup of static electricity [18]. The SMS hood fabric is designed for single-use and not intended for reuse, as they may start to tear and fail but the rest of the parts can be disinfected and reused.

Respirator systems that are helmet-based are proven to be superior to the facemask-based systems. Studies have been conducted in ventilating patients with Acute Respiratory Distress Syndrome like the ones caused by COVID-19 disease. Clinical trial of helmet versus facemask noninvasive ventilation (NIV) have shown that the patients who have undergone helmet based NIV exhibited a greater rate of recovery and functional independence [19], [20]. Though PROPER may not be an equipment for NIV, these studies show the advantage of a helmet-based respirator system.

A helmet fitting (3 M™ Peltor™ G2N Steering adjust to G2000 o G3000) with an adjustable ratchet knob at the back of the head is fitted inside the hood to provide some structure to the hood and prevent the weight of the breathing tube from pulling the hood. A small hole of 25 mm in diameter is cut in the hood fabric to allow the ratchet knob to be placed outside to reach with hand while donning the hood. A 3D printed adaptor extruding out of the hood in the rear side through a 30 mm in diameter hole cut in the hood fabric just below the hole for the ratchet knob, acts as an interface to attach one end of the breathing tube. The adaptor is secured to the hood with a flange fitted in the exterior of the hood (see Fig. 3).

The 3D printed curved face shield frame acts as the visual interface for the operator wearing the PROPER hood. A transparent sheet (OHP film) is secured to the frame with the help of a double-sided white foam tape (Tesa 62936) to achieve an airtight seal and clear visibility to the surrounding. The frame with its wider field of view of greater than 150° provides a comfortable vision for the operators.

The resulting environment is completely sealed inside the hood and exhibits closely the characteristics of a cleanroom. It is therefore possible to quantify its ISO class cleanliness level by measuring the count of 0.3 µm size particles inside the hood. Keeping the number of particles low is an extremely hard task but monitoring this number and keeping it under a threshold value by using filters and cleaning maintains an enclosed environment pristine from external contamination.

### 2.2 Fan filter unit (FFU)

Typically, a Fan Filter Unit (FFU) is used in cleanroom constructions. The basic working principle of a FFU entails a static pressure fan placed against an air purification filter to pass the purified air at a specific flow. The choice of the air purification filter depends on the type of particles that the user wants to trap. Filters with high efficiency at 0.3 µm particle size can in theory trap particles down to the size of the SARS-CoV-2 virus (approximately 0.12 µm in diameter) containing aerosols. For PROPER, we designed a miniaturized FFU. PROPER uses a corrugated HEPA filter cartridge (Nilfisk HEPA H14 CPL) normally used for vacuum cleaners, that is 99.97% efficient at trapping and retaining particles down to and including 0.3 µm at a specific air velocity. The HEPA filter captures the particles bigger than 1 µm on its fibers, particles between 0.3 µm and 1 µm through interception, and for particles <0.3 µm through Brownian diffusion [21]. However, the fractional capture efficiency decreases with increasing air velocity and increases rapidly below 0.1 µm and above 0.3 µm. It is important to point out that the particles of the size of a virus such as SARS-CoV-2 are the most difficult to capture. The fan used in PROPER creates furthermore an overpressure within the environment, which impedes the uncontrolled inflow of air from the exterior (at lower pressure) to the inside (at higher pressure). Table 1 shows the types of filters used in healthcare and their capture efficiency and penetration levels. HEPA filters clearly outperform the other widely used N95, FFP2 or FFP3 filters in healthcare settings.

**Table 1:** Comparison of capture efficiency of masks and HEPA filters [4].

Masks and Filter	Capture efficiency	Penetration levels
N95 mask (NIOSH)	>95%	<5%
FFP2 mask (EU)	>94%	<6%
FFP3 mask (EU)	>99%	<1%
HEPA filter in PAPR (US DOE)	>99.97% (depends on air velocity and particle size)	<0.03%

**Fig. 2:**
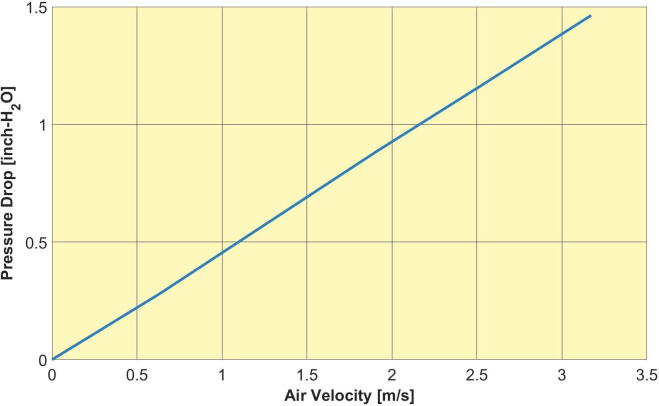
Pressure drop as a function of air velocity of the HEPA filter used in PROPER.

PROPER also uses an additional 10 mm thick Urethane foam activated carbon prefilter (RS Pro) that can absorb 80% of noxious flux and lead fume particles. Since the HEPA filter is in the pathway of air, a resistance in the flow of air is intrinsic. As the air flows through the HEPA filter, a drop in static pressure is observed across the filter that depends on the air flow. Hence, to achieve a specific air flow at the end of the PAPR system tubing into the hood, it is important to consider a fan with a static pressure higher that the cumulative pressure drop across the prefilter, HEPA filter and through the tubing. The static pressure drop of the HEPA H14 filter used in PROPER is unknown due to lack of technical specifications of the product. But similar filters with glass fiber media such as AireFlow-HC from Airepure Australia Pty Ltd [22] and V-bank series from Rainbow filters [23] are designed to operate in systems with air velocities of 2.54 m/s and a pressure drop of 1.17 in.-H_2_O. Fig. 2 shows the pressure drop as a function of air velocity for the HEPA filter used. Hence, while choosing a fan, we assumed a total static pressure drop of 1.5 in.-H_2_O across prefilter, HEPA H14 filter and through 3D printed parts and tubing, meaning the fan must deliver a static pressure of at least 1.5 in.-H_2_O while delivering an air flow of 6–8.8 CFM (170–250 LPM) which is typical for loose-fitting PAPR systems [24], [25].

**Fig. 3:**
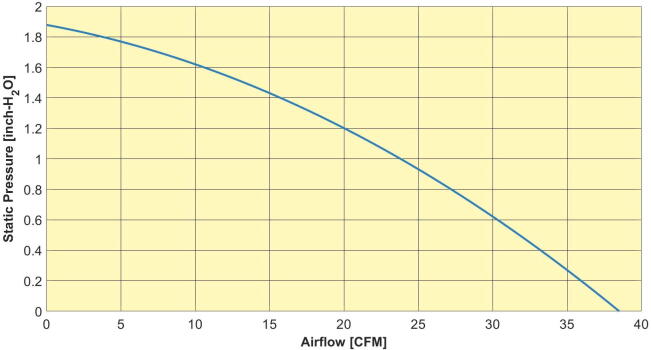
Characteristic curve of the centrifugal fan showing the performance of static pressure vs air flow as reported in the datasheet.

The centrifugal fan is the main driver of the FFU. Centrifugal fans are capable of generating relatively high pressures and are suitable for high pressure applications as compared with axial flow fans. The air enters the impeller in an axial direction and is discharged at the impeller outer periphery. The air flow moves along the centrifugal direction (or radial direction) [26]. PROPER uses a 12 VDC, 19.2 W radial type centrifugal fan (NMB Technologies, 09533GA-12Q-AA-00) with a maximum static pressure of 1.88 in.-H_2_O and a maximum air flow of 38.1 CFM. Fig. 3 shows the characteristic curve representing the static pressure vs air flow performance of the chosen centrifugal fan.

For the assumed total static pressure drop of 1.5 in.-H_2_O across prefilter, HEPA H14 filter and through 3D printed parts and tubing, the fan is capable of delivering a maximum air flow of about 13 CFM. Furthermore, the fan can operate between 9.5 V and 13.2 V efficiently and hence by changing the voltage, the motor speed can be varied and therefore the air flow can be controlled. To provide an airtight seal in the pathway between the centrifugal fan to the 3D printed adaptor where the other end of the breathing tube is attached, the whole FFU assembly with the prefilter, centrifugal fan, HEPA H14 filter and the adaptor are made into one complete assembly interfaced with 3D printed components.

### 2.3 Breathing tube

The breathing tube reliably connects the adaptor in the hood to the adaptor in the FFU to ensure an unrestricted flow of purified breathing air. PROPER uses a one-meter long flexible breathing tube (3M, BT-20L) that is typically used with 3 M™ Versaflo™ PAPR systems. Since the air passing through the breathing tube is in direct contact with the operator’s respiratory system, the product must be FDA certified and this model complies with that requirement and simplifies the validation process. This modular approach not only simplifies the equipment design process but is also more convenient to allow for replacement of pieces, and for maintenance and cleaning of the hood and FFU units.

### 2.4 Power supply and conditioning unit

The only power consuming unit of the entire PROPER assembly is the centrifugal fan in the FFU. An uninterrupted DC source providing a constant voltage between 9.5 V and 13.1 V is achieved with a combination of a Lithium Ion (rechargeable) powerbank (Linocell, 15,600 mAh) and an adjustable DC-DC step-up module (Velleman, LM2577). The powerbank is a constant 5 V DC source that can deliver a total of up to 3 A with a charging time of 9 h. The 5 V, 3 A DC power source is converted to a 12 V, 2 A (temporary 3 A) power supply via the LM2577 voltage converter booster of the adjustable DC-DC step-up module. A tiny screw potentiometer helps with tuning the voltage to the desired value. Under nominal operation, PROPER consumes a power of about 5 V, 1.6 A.

### 2.5 Safety features

The powerbank has an inbuilt Low Voltage Disconnect (LVD) function that prevents the battery from deep discharging. The LVD functions by sensing the threshold voltage and cuts off the power delivery upon reaching the threshold. In critical applications, such as the PPE donned by the HCP in risky environments, the LVD function is complementary to a battery level indicator warning the operator. The powerbank also has an inbuilt battery voltage level indicator which is displayed with a series of four blue LEDs where all four LEDs glowing represents fully charged and no LED glowing represents fully discharged state. However, it is safe to exit the risk area when the battery voltage level indicator has only one LED glowing as a precaution.

Another additional safety feature in case of shorting of the circuits is established with a fuse. A slow blow glass tube fuse that fails when the current exceeds 2 A is used in PROPER to avoid this problem.

### 2.6 Disposal and disinfection

With the exception of the SMS hood fabric and the HEPA filter cartridge which, as any other filter, may allow limited reuse, it is recommended to disinfect and reuse almost all the other components of PROPER assembly. When disposing the hood fabric, adequate care must be taken to isolate it as a critical biohazard waste. Upon a review of all the recommended methods of disinfecting 3D printed components [5], [27], heat treatment is not a viable option since the glass transition temperature of Polylactic Acid (PLA) plastic is between 60 and 65 °C and precludes the use of either alcohol or hypochlorite solutions. Application of detergent based treatments seems promising in terms of efficiency. However, we suggest to 3-D print the parts using Polyethylene terephthalate glycol (PETG) for its wide availability of disinfection methods as described in [27]. The detailed procedures for disposal and disinfection of individual components of PROPER assembly is discussed in “Operation Instructions” section.

## 3 Design files

The source to the design files can be found in the Mendeley repository > Data > Design Files. Table 2 specifies the design file name, file type and type of open source license of each part.

**Table 2:** Design Files Summary.

Where the names correspond to: a) Curved_Face_Shield_Frame, is the CAD file of the 3D printed face shield frame sealing the open cutting in the hood; b) Hood_Adaptor, is the CAD file of the 3D printed adaptor connecting one end of the breathing tube in the hood side; c) Hood_Adaptor_Flange, is the CAD file of the 3D printed flange used to secure the Hood_Adaptor with the hood; d) Breathing_Tube_Adaptor, is the CAD file of the 3D printed adaptor connecting other end of the breathing tube in the FFU side; e) HEPA_Filter_Inlet_Adaptor, is the CAD file of the 3D printed adaptor attached to the inlet side of the HEPA filter cartridge; f) HEPA_Filter_Outlet_Adaptor, is the CAD file of the 3D printed adaptor attached to the outlet side of the HEPA filter cartridge; g) Right_Angle_Duct, is the CAD file of the 3D printed right angled duct interfacing the HEPA_Filter_Inlet_Adaptor and the centrifugal fan and h) Prefilter_Holder, is the CAD file of the 3D printed prefilter holder in front of the centrifugal fan.

**Print configuration**

The following print configuration was used to print all the 3D printed parts mentioned above:

Printer: Ultimaker 3
Material: PLA, Ø 2.85 mm; For the purpose of demonstration we have used here PLA, however, PETG is recommended for easier disinfection purposes.
Print settings: 0.8 mm nozzle, 0.2 mm layer height, 50% infill density, Support enabled

## 4 Bill of Materials

The cost of each unit is about 180 GBP plus 5 GBP for the cleanroom garment, which should be bought for every new use (see Table 3).

**Table 3:** Bill of Materials of PROPER.

## 5 Build Instructions

This instruction guide provides a visual overview of the assembly actions required to build a PROPER unit. Each component has been assigned a designator code with the nomenclature in the Bill of Materials. We recommend performing the building procedure in a cleanroom of at least ISO 5 standards in a proper cleanroom garment with mask and gloves, to prevent contamination of the components that may not be filtered later on once the assembly is over. A series of instruction video tutorials for building PROPER is also enclosed in the Mendeley repository.

### 5.1 Hood

**Fig. 4:**
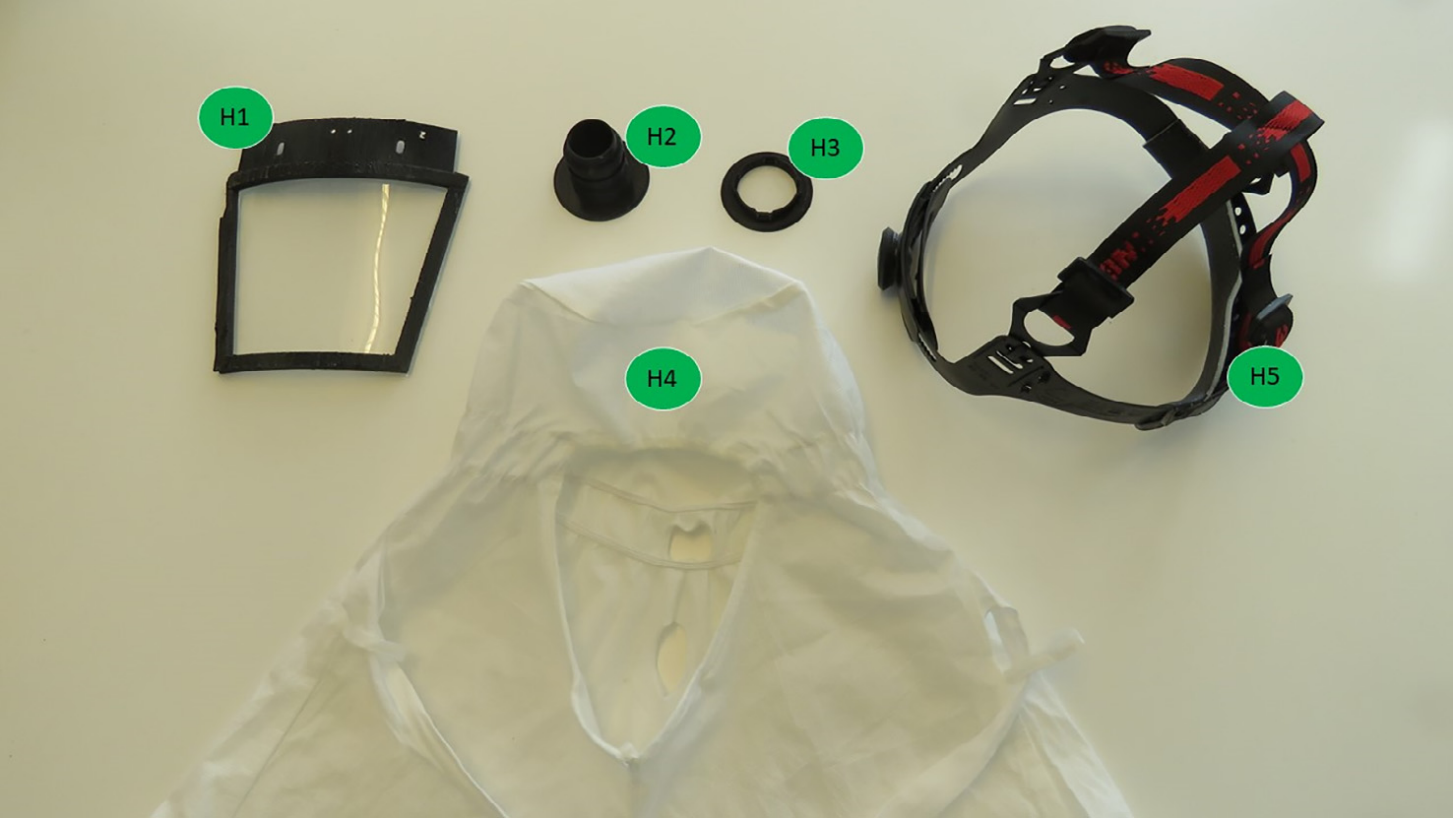
Components for building the hood are denoted by their designator codes. H1 – Curved face shield frame, H2 – Hood adaptor, H3 – Hood adaptor flange, H4 – SMS hood fabric, and H5 – Helmet fitting.

Fig. 4 shows the components to build the PROPER hood assembly where each component has its individual designator code. Some of these components are 3D printed prior to this procedure.

Step 1: Attaching the curved face shield frame to the helmet fitting

**Fig. 5:**
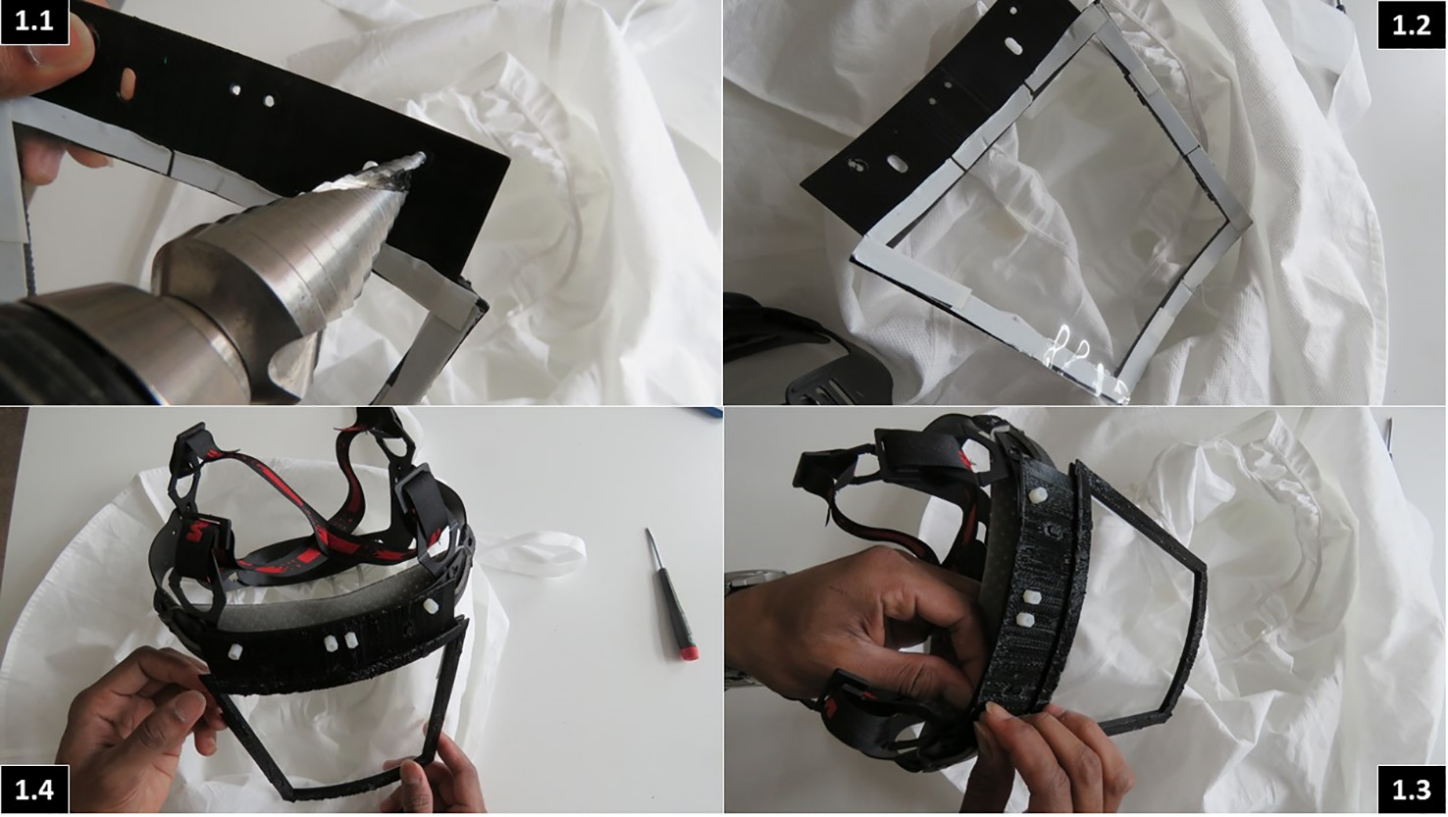
(Clockwise) Procedure to drill holes for attaching the curved face shield frame to the helmet fitting.

Position the inward curve face of the 3D printed curved face shield frame (H1) towards you and line its frame edges with a 5 mm wide double-sided white foam tape (M2). Cut a transparent sheet (OHP film) to the desired dimensions to align with the outer edges of the curved face shield frame (H1). Place the cut transparent sheet over the lined double-sided white foam tape (M2) and gently dab to ensure proper adhesion between the transparent sheet and the curved face shield frame (H1) as shown in Fig. 5 (top right).

Align the curved face shield frame (H1) with the helmet fitting (H5) so that the two oval slots match each other in both components. Drill at least four M3 holes in the curved face shield frame (H1) while aligning with the holes in the helmet fitting (H5) as shown in Fig. 5 (top left). Try to make sure these holes are evenly distributed throughout the area of the curved face shield frame (H1) as shown in Fig. 5 (top right).

Use a M3 bolt and nut to secure the curved face shield frame (H1) and helmet fitting (H5) through the drilled holes as shown in Fig. 5 (bottom right) and Fig. 5 (bottom left). Make sure that the other side of the screw does not project too much on the head side that may lead to injuries while tightening the helmet fitting (H5) with the head. When fabricating the units that shall be used in clinical environments, these manipulations should be done with sterile gloves and/or clean hands (using a hand sanitizer).

Step 2: Providing structure to the hood fabric with the helmet fitting and attaching the face shield

**Fig. 6:**
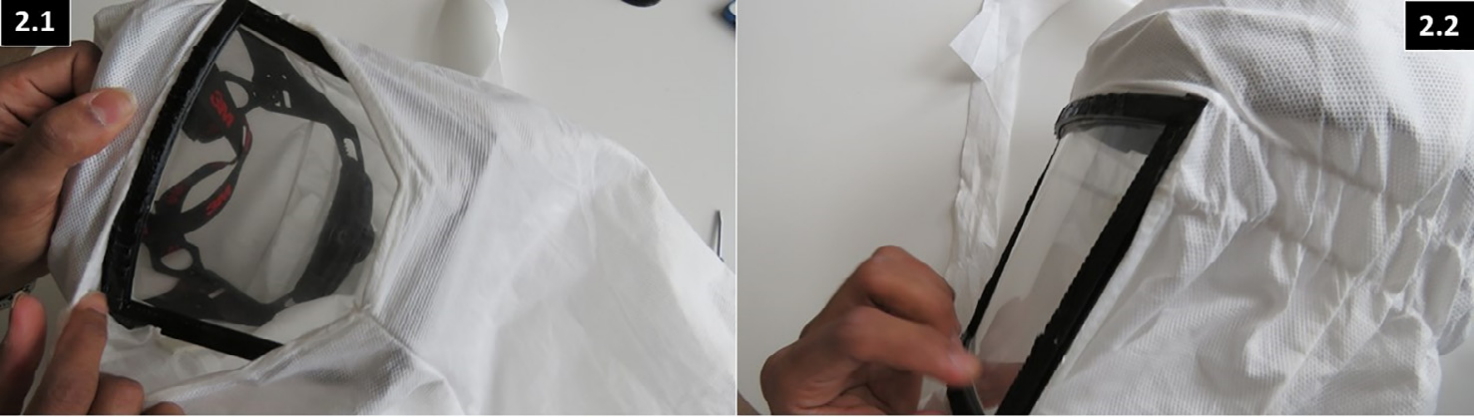
(Left to right) Procedure to attach the curved face shield frame and helmet fitting to the SMS hood fabric.

Open the bottom part of the hood fabric (H4) and place the assembly of the curved face shield frame (H1) and helmet fitting (H5) inside. Gently place the helmet fitting (H5) to the top of the hood fabric (H4) and curved face shield frame (H1) toward the open cutting in the hood fabric (H4). Slowly pull the beaded edges of the open cutting in the hood fabric (H4) and insert it in the slot along the sides of the curved face shield frame (H1) as shown in Fig. 6 (left) and 6 (right). You may use a small blunt object to insert the fabric properly into the slot on all four sides. This procedure ensures a good airtight seal in front of the operator’s face.

Step 3: Securing the ratchet knob and hood adaptor

**Fig. 7:**
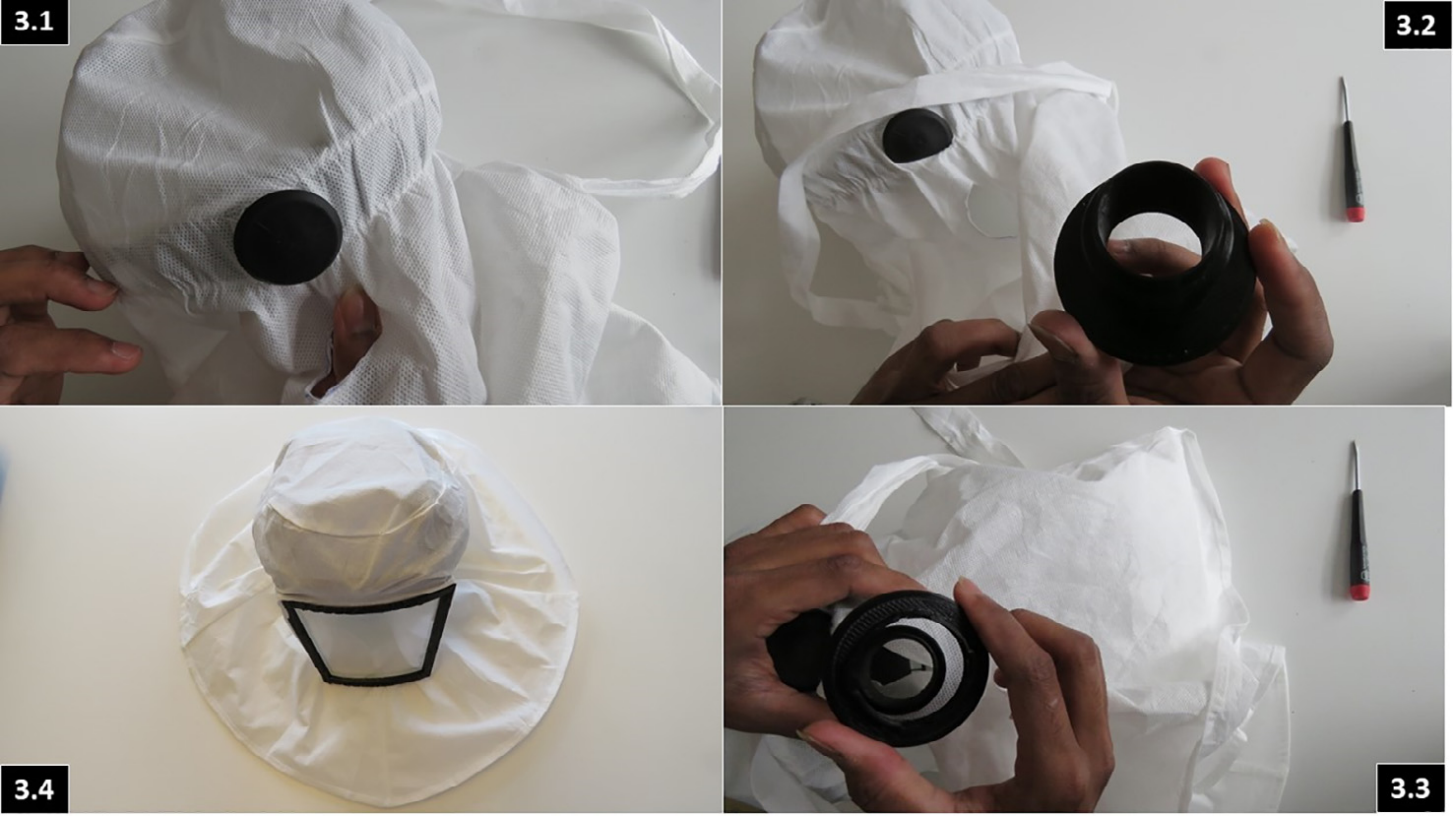
(Clockwise) Procedure to secure the ratchet knob and hood adaptor. (bottom left) Completed PROPER hood assembly.

Now that the curved face shield frame (H1) and helmet fitting (H5) is secured in place inside the hood fabric (H4), gently pull the hood fabric (H4) while holding the helmet fitting (H5) to allow the ratchet knob into the 25 mm hole that was cut at the back side of the head as shown in Fig. 7 (top left). Be slow and patient while stretching the fabric around the knob as this operation may tear the fabric and leave an air gap if it is performed too fast.

Insert the 3D printed hood adaptor (H2) from the inside of the hood fabric in the 30 mm in diameter hole that was cut just below the hole for ratchet knob as shown in Fig. 7 (top right). From the outside of the hood fabric (H4) secure the hood adaptor (H2) with the 3D printed hood adaptor flange (H3) as shown in Fig. 7 (bottom right). This procedure provides the secure pathway to connect the breathing tube to the hood assembly as shown in Fig. 7 (bottom left).

### 5.2 Fan filter unit (FFU)

**Fig. 8:**
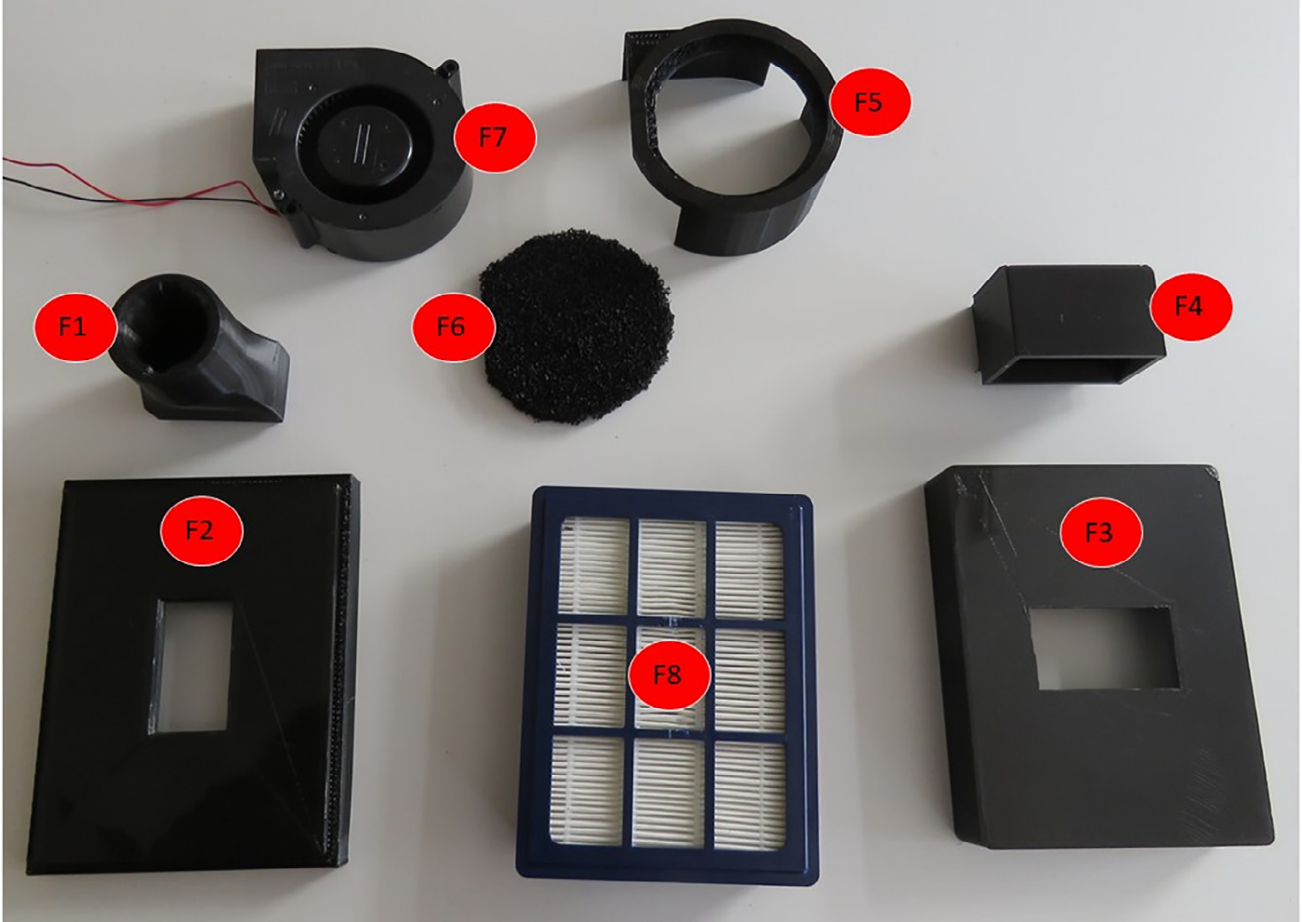
Components for building the Fan Filter Unit (FFU) are denoted by their designator codes. F1 – Breathing tube adaptor, F2 – HEPA filter outlet adaptor, F3 – HEPA filter inlet adaptor, F4 – right angle duct, F5 – Prefilter holder, F6 – Prefilter, F7 – Centrifugal fan, and F8 – HEPA H14 filter.

Fig. 8 shows the components to build the miniaturized Fan Filter Unit (FFU) with each component assigned an individual designator code. Some of these components are 3D printed prior to this procedure.

Step 4: Enclosing the HEPA filter inside the adaptors to create an airtight assembly

**Fig. 9:**
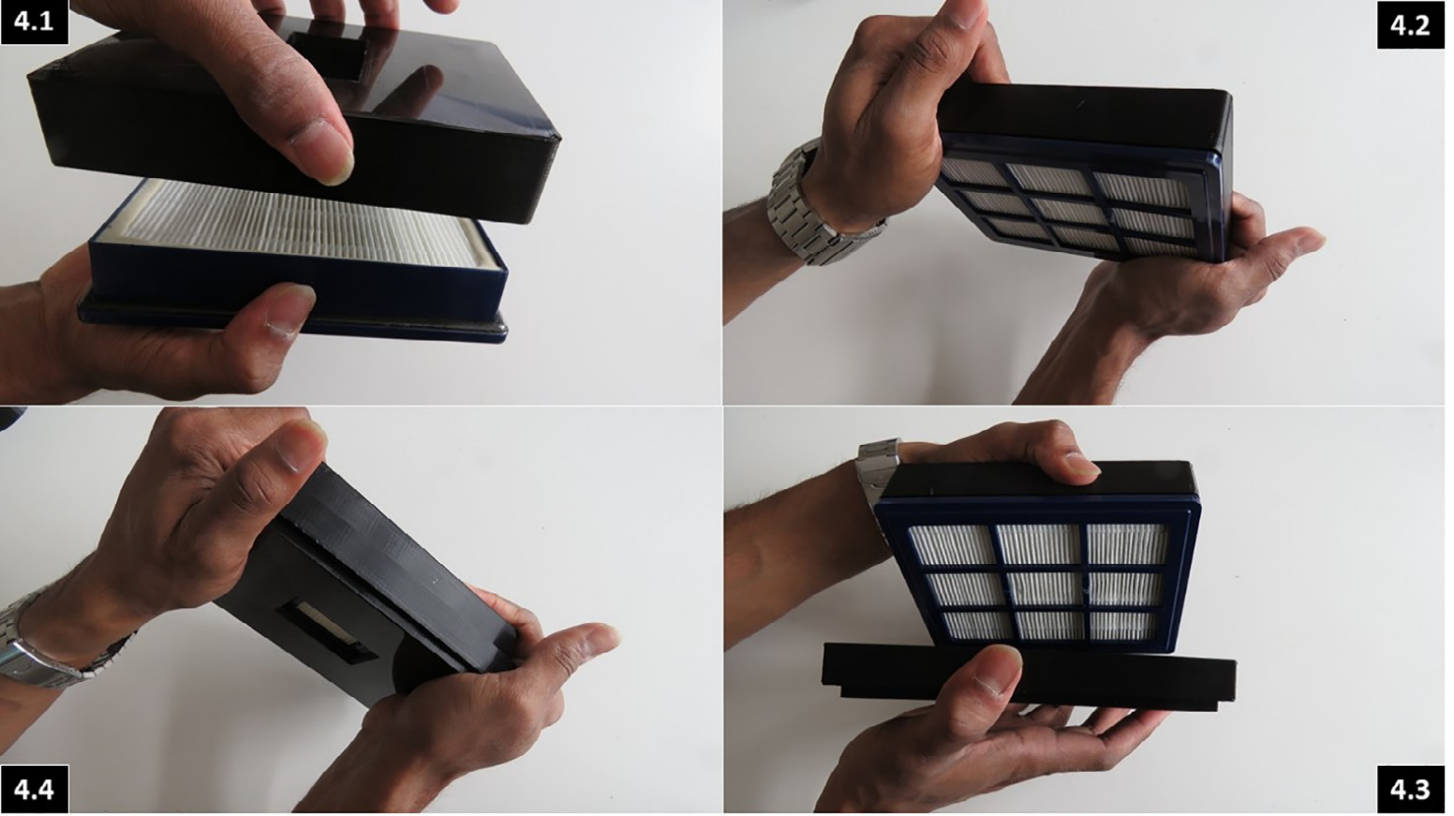
(Clockwise) Procedure to enclose the HEPA filter inside the adaptors to create an airtight assembly.

Place the shorter side of the HEPA 14 filter (F8) aligning the edges of the 3D printed HEPA filter inlet adaptor (F3) as shown in Fig. 9 (top left). Press firmly to hear a click to fit the HEPA H14 filter (F8) complete inside the HEPA filter inlet adaptor (F3) as shown in Fig. 9 (top right).

Now, place the longer side of the HEPA 14 filter (F8) aligning the edges of the 3D printed HEPA filter outlet adaptor (F2) as shown in Fig. 9 (bottom right). Again, press firmly to hear a click to fit the HEPA H14 filter (F8) and the HEPA filter inlet adaptor (F3) inside the HEPA filter outlet adaptor (F2) as shown in Fig. 9 (bottom left). This procedure completes the building of the filtration unit.

Step 5: Securing the air pathway with adaptors and attaching the centrifugal fan

**Fig. 10:**
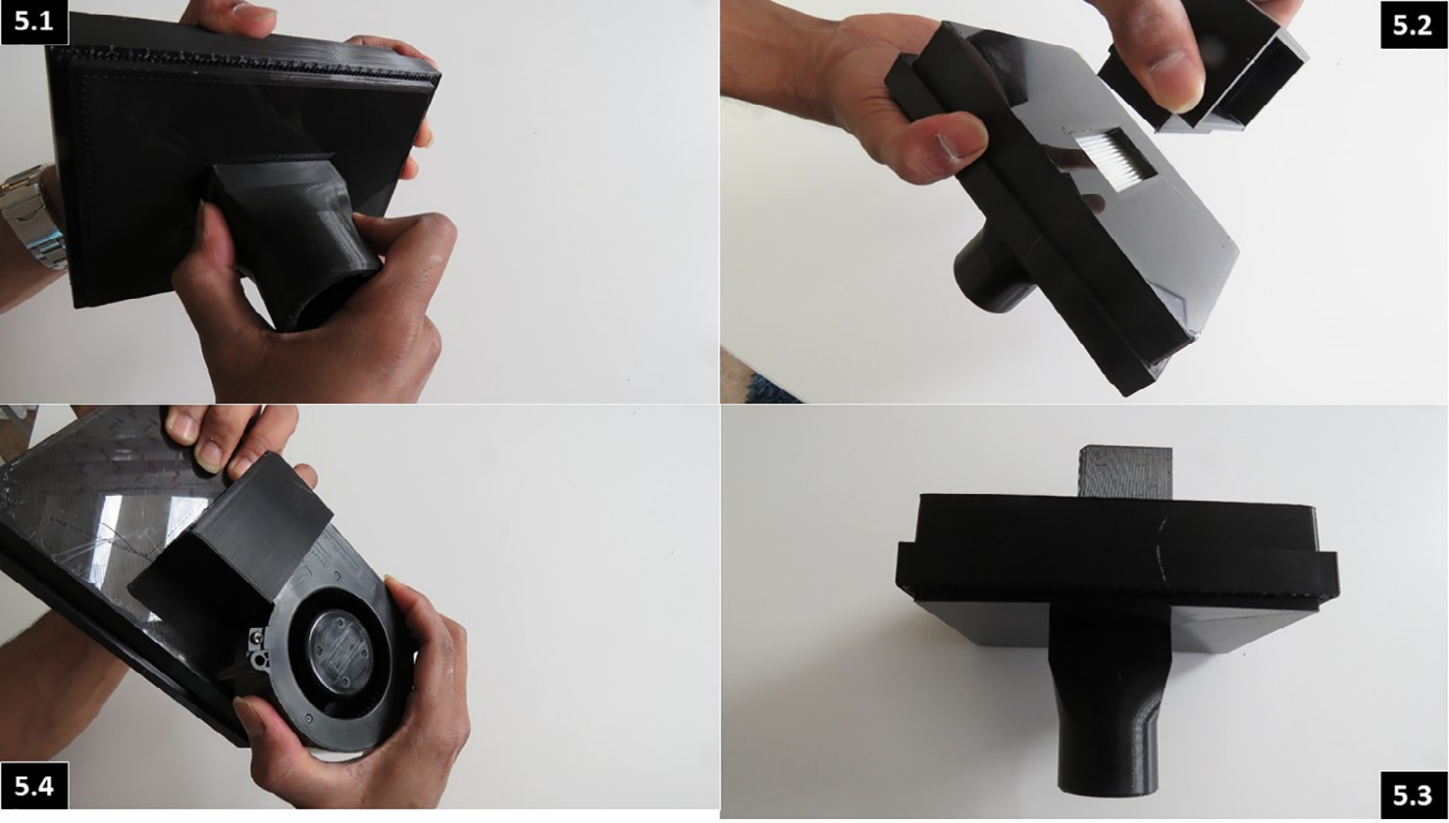
(Clockwise) Procedure to secure the air pathway with adaptors and attaching the centrifugal fan.

Attach the 3D printed breathing tube adaptor (F1) to the HEPA filter outlet adaptor (F2) as shown in Fig. 10 (top left). Similarly, attach the 3D printed right angle duct (F4) to the HEPA filter inlet adaptor (F1) as shown in Fig. 10 (top right) such that the completed filtration unit resembles Fig. 10 (bottom right).

Insert the centrifugal fan (F7) to the opening in the right-angle duct (F4) such that the open suction side is facing the open air. You may push the centrifugal fan (F7) inside the duct to the point that the curved face is in contact with the duct edges as shown in Fig. 10 (bottom left). This procedure makes sure that the air pathway is properly secured and sealed.

Step 6: Securing the prefilter in front of the centrifugal fan

**Fig. 11:**
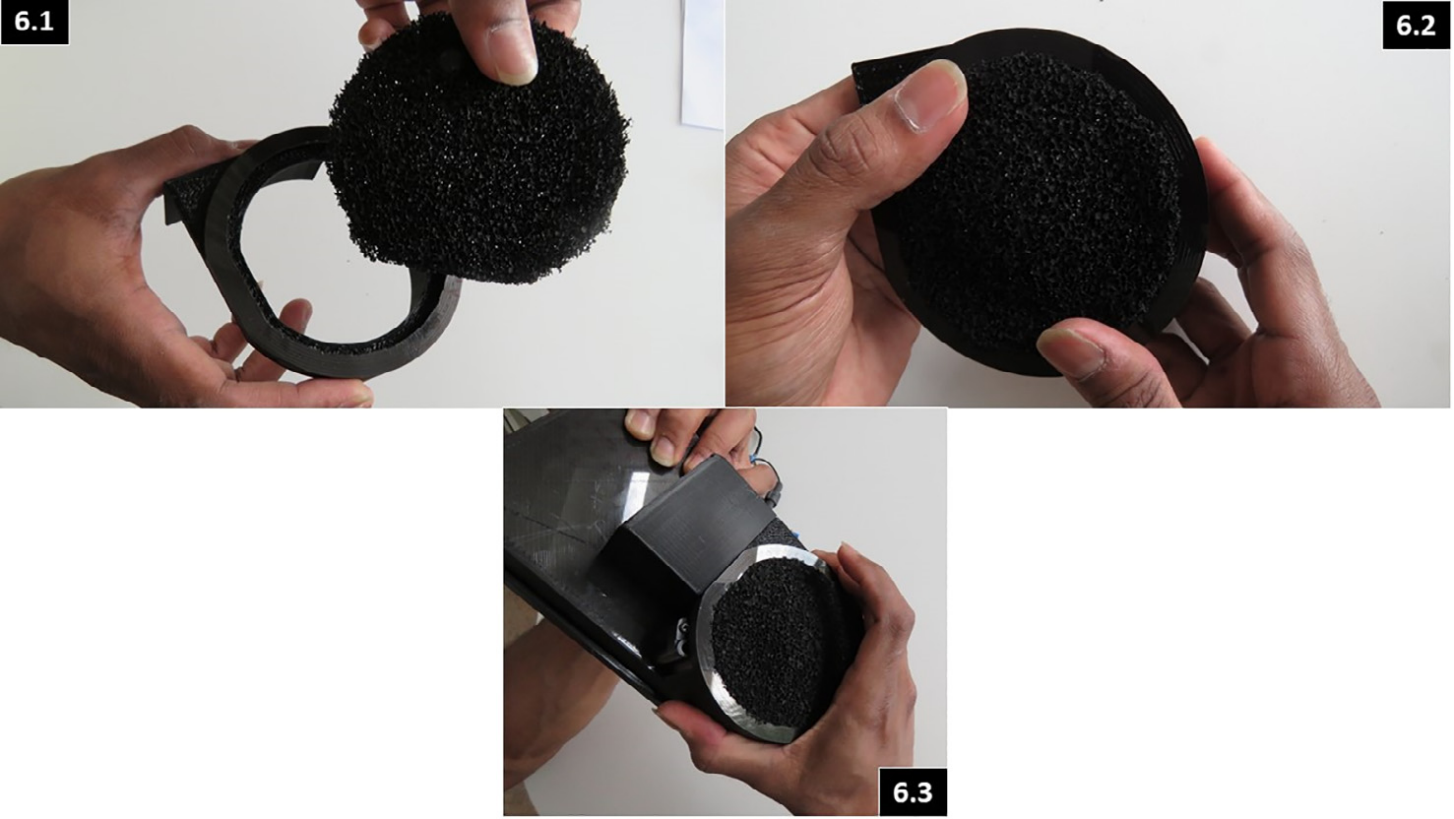
(Clockwise) Procedure to secure the prefilter in front of the centrifugal fan.

Take a sheet of the prefilter (F6) and cut it into a 93.5 mm in diameter circle with a cropped edge at 5 mm distance from any point along the circumference of the circle as shown in Fig. 11 (top left). Gently place the cut prefilter (F6) on the prefilter holder (F5) and push it along its circumference to firmly attach it inside the slot as shown in Fig. 11 (top right). Now, secure the prefilter holder (F5) along with the attached prefilter (F6) in front of the centrifugal fan (F7) such that the open suction side of the fan is facing against the prefilter as shown in Fig. 11 (bottom center). This procedure ensures that the air that is entering the centrifugal fan (F7) is free of smoke particles.

### 5.3 Power supply and conditioning unit

**Fig. 12:**
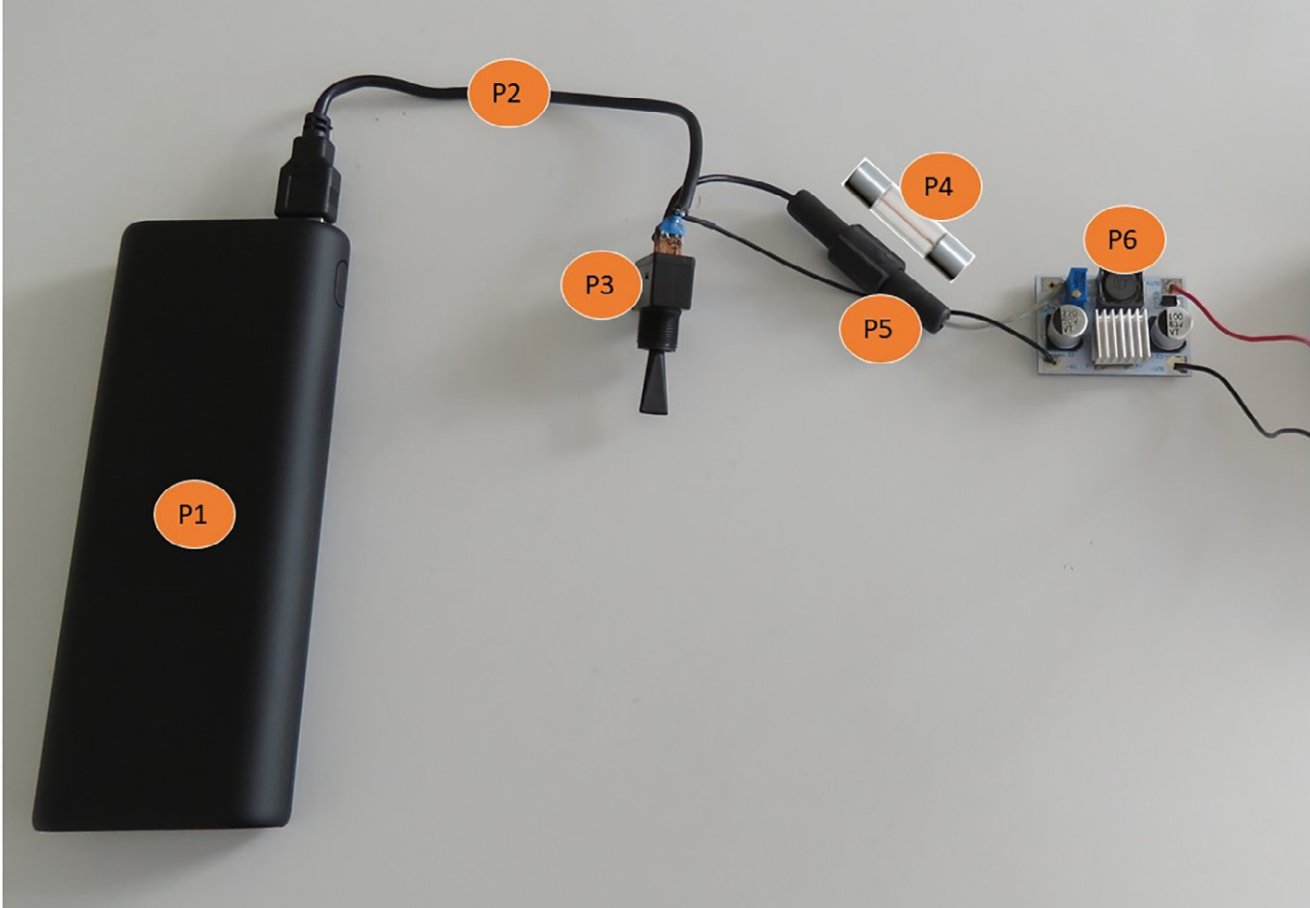
Components for constructing the power supply and conditioning unit are denoted by their designator codes. P1 – Powerbank, P2 – USB charging cable, P3 – Switch, P4 – Fuse, P5 – Fuse holder, and P6 – Adjustable DC-DC step-up module.

Fig. 12 shows the components to build the power supply and conditioning unit with each component assigned an individual designator code. You would need a solder station and lead for this build procedure.

Step 7: Constructing the power supply and conditioning unit

Cut the USB charging cable (P2) closer to the barrel end and sleeve off the cable insulation to expose the supply and ground wires, typically color-coded in red and black respectively. Make sure that the end of black wire is at least 10 cm longer than the end of red wire. Turn ON the solder station to heat the solder iron to a predefined temperature (typically 400 °C) and solder the supply (red) wire to the first terminal of the switch (P3).

Place the fuse (P4) inside the fuse holder (P5) and lock it. Solder the wire at one end of the fuse holder (P5) to the second terminal of the switch (P3).

Solder the wire at the other end of the fuse holder (P5) to the positive input terminal of the adjustable DC-DC- step-up module (P6). Also, solder the black wire of the USB charging cable (P2) directly on to the negative input terminal of the adjustable DC-DC- step-up module (P6). This procedure yields the power conditioning circuit.

Solder the red wire of the centrifugal fan (F7) to the positive output terminal of the adjustable DC-DC- step-up module (P6). Also, solder the black wire of the centrifugal fan (F7) to the negative output terminal of the adjustable DC-DC- step-up module (P6). The input and output, positive and negative terminal in the adjustable DC-DC- step-up module (P6) are printed in the breakout board.

Connect the USB-A end of the USB charging cable (P2) in the above power conditioning circuit to the USB-A slot in the powerbank (P1) that acts as a power supply. This procedure completes the construction of the power supply and conditioning unit. You may switch ON the unit to observe if the fan is operating as expected.

### 5.4 Hip bag assembly

Step 8: Securing the adjustable DC-DC step-up module

**Fig. 13:**
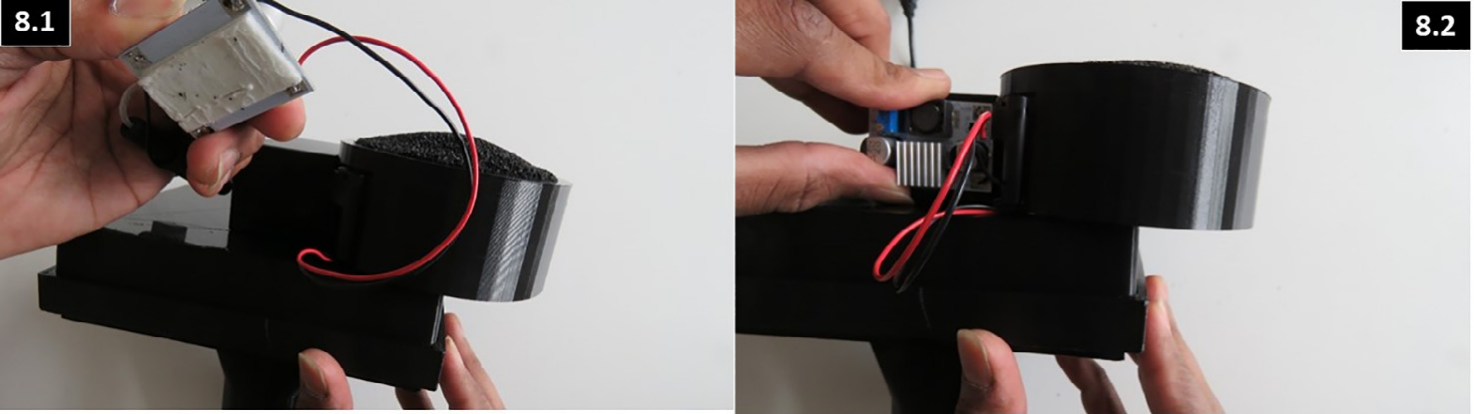
(Left to right) Procedure to secure the adjustable DC-DC step-up module.

Cut a small piece of the double-sided white foam tape (M2) and place under the Adjustable DC-DC step-up module (P6) as shown in Fig. 13 (left). Attach the bottom of the Adjustable DC-DC step-up module (P6) over the 20 mm in diameter hole on the right-angle duct as shown in Fig. 13 (right). This hole allows a little bit of backflow of air out and hence serves as a natural cooling system for the adjustable DC-DC step-up module (P6) that may produce heat during its operation for prolonged usage. This procedure secures the module within the entire assembly.

Step 9: Placing the FFU and power supply and conditioning unit inside the hip bag

**Fig. 14:**
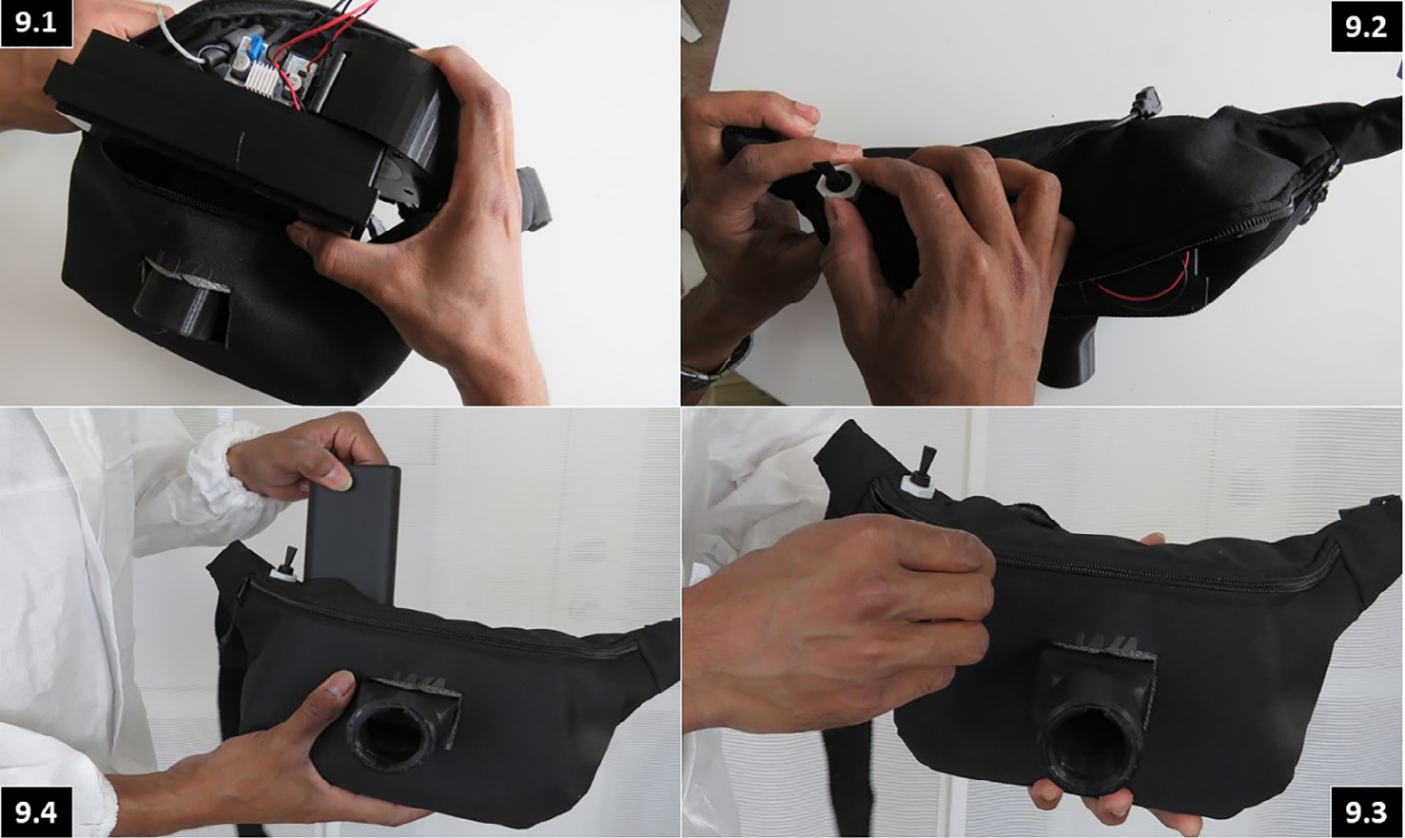
(Clockwise) Procedure to place the FFU and power supply and conditioning unit inside the hip bag.

Cut a square hole of dimensions 55 mm wide and 30 mm high in the front side of the hip bag (M1). Also, cut a circular 60 mm in diameter hole in the back of the hip bag (M1). Now, place the entire FFU and power supply and conditioning unit inside the hip bag (M1) such that the breathing tube adaptor (F1) is aligned with the square hole and the prefilter folder (F5) and prefilter (F6) are aligned to the circular hole as shown in Fig. 14 (top left).

Make a small 5 mm in diameter hole in the top-left side of the hip bag (M1) and secure the switch (P3) with a nut on the outside as shown in Fig. 14 (top right).

Cut a rectangular hole of dimensions 60 mm wide and 15 mm high in the top-left side of the hip bag (M1) next to the hole for the switch (P3). Leave out the USB charging cable (P2) through this rectangular hole.

Close the zip of the hip bag (M1) as shown in Fig. 14 (bottom right). Place the powerbank (P1) in the rectangular hole vertically inside the hip bag (M1) as shown in Fig. 14 (bottom left). This procedure completes the hip bag assembly with FFU and power supply and conditioning unit inside that can always be worn around the hip during PROPER operation.

Step 10: Completing the power supply and conditioning unit

**Fig. 15:**
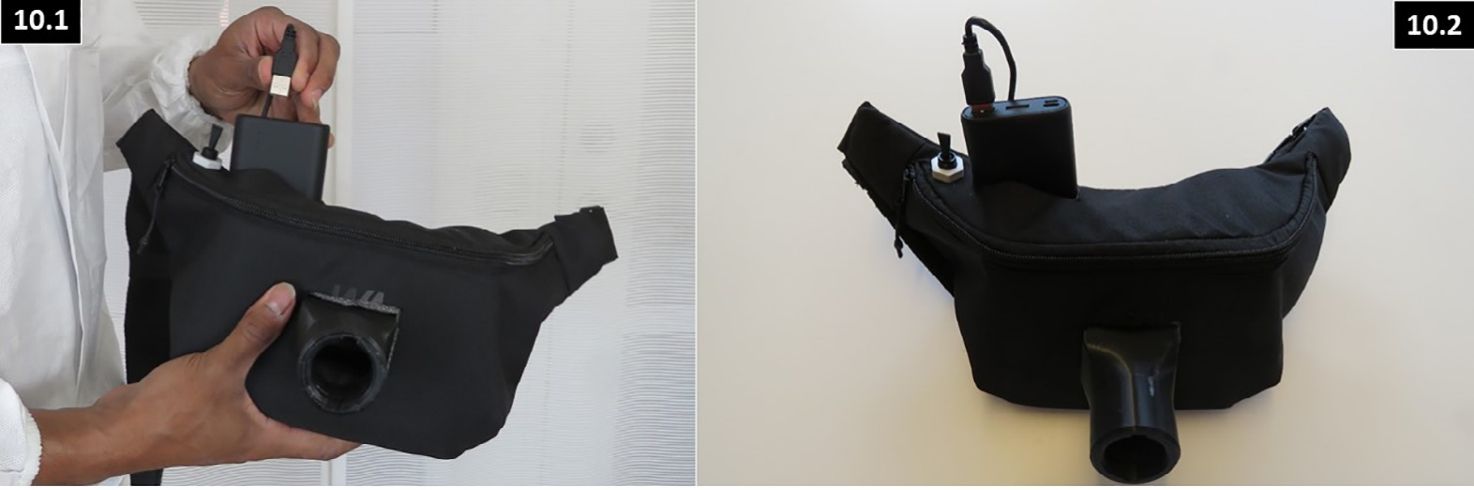
(Left to right) Procedure to complete the power supply and conditioning unit.

Connect the USB-A end of the USB charging cable (P2) that is left out of the hip bag through the rectangular hole to the USB-A slot in the powerbank (P1) as shown in Fig. 15 (left). This procedure completes all the electrical connections in the power supply and conditioning unit and readies the PROPER unit for operation. Remember to fully charge the powerbank (P1) before every operation.

### 5.5 Interfacing the pieces

Step 11: Connecting the breathing tube between the hood and the FFU

**Fig. 16:**
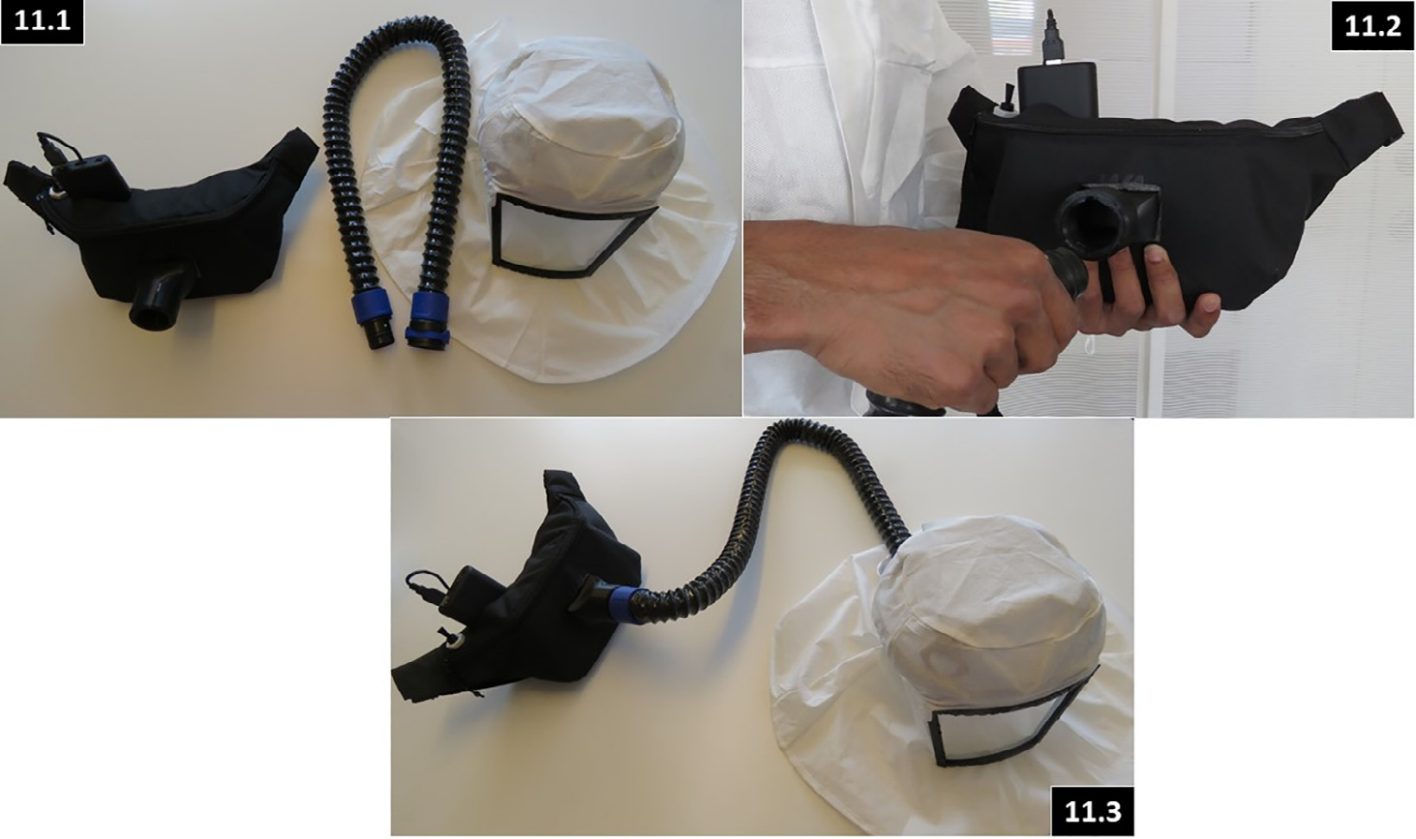
(Clockwise) Procedure to connect the breathing tube between the hood and the FFU.

Now that the hood, FFU, and the power supply and conditioning unit are individually assembled and accommodated in the hip bag, the two pieces, hood and hip bag can be interlinked via the breathing tube between the hood adaptor (H2) on the hood side and the breathing tube adaptor (F1) on the hip bag side (see Fig. 16).

## 6 Operation Instructions

### 6.1 Donning the PROPER

The procedure for donning the cleanroom garment has been described in accordance with the guidelines stated by the Centers for Disease Control and Prevention (CDC) of United States [28], [29].

**Table:** 

Step 1: Donning the PROPER begins with a hand hygiene procedure and putting on the cleanroom garment. The steps are briefed as follows: 2. The cleanroom garment must be chosen based on the correct size. 3. Hand hygiene needs to be ensured using a hand sanitizer. 4. Cleanroom garment is worn with all the ties on the garment secured. Assistance may be needed by other healthcare professional for securing the ties.	
Step 2: Once the cleanroom garment has been worn, the hood needs to be placed on the head.	
Step 3: The shroud of the hood is inserted under the overall garment and zipped up.	
Step 4: The ratchet knob is tuned by rotating in clockwise direction and anti-clockwise direction ensuring that the PROPER hood is firmly secured to the head.	
Step 5: The hip bag is secured around the hip using the strap and buckle. Ensure that the hip bag is secured in a comfortable manner.	
Step 6: Connect the breathing tube to the hood adaptor. A click sound ensures that the connection is perfectly sealed.	
Step 7: The PROPER fan unit can now be powered ON.	
Step 8: The PROPER unit will reach the maximum air flow in about 5 s after turning ON. The user is now enclosed within an ISO 6 area and can enter a potentially dangerous area such as an intensive care unit.	

### 6.2 Doffing the PROPER

After exiting the ICU, the doffing procedure is carried out in a dedicated isolated environment. The gloves are first removed and disposed before beginning the doffing. The procedure for doffing is followed exactly in the reverse order of the donning steps. Once the doffing is completed, the single use components need to be disposed safely and the components that can be disinfected must be disinfected before next use. The disposal and disinfection procedure are discussed in the next section.

### 6.3 Disposal and disinfection procedure

After operating in a relevant environment, SARS-CoV-2 virus causing acute respiratory infections or other airborne pathogens can survive on PROPER components for variable periods of time, from hours to weeks. Consequently, contaminated PROPER must be handled, cleaned, and disinfected properly to reduce the possibility of the equipment contributing to disease transmission. In general, cleaning and disinfecting consists of dissembling the PROPER (following the procedure in “Build Instructions” section in reverse order), cleaning and disinfecting components, thoroughly rinsing components, and reassembling the PROPER when components are dry. Cleaning is recommended after each use, but the PROPER must be cleaned as often as necessary to prevent them from becoming unsanitary. Only the SMS hood fabric and HEPA filter cartridge of PROPER must be discarded after each use and isolated as critical biohazard waste. We recommend performing the disinfection procedure in an isolated environment with proper overall garment with mask and gloves, to limit the potential for self-infection.

Based on the CDC’s recommendations on cleaning and disinfection of PAPR components [5] and Prusa face shield disinfection methods [27], several recommended methods exist that were tested by leading laboratories to ensure conclusive and reliable results. We recommend cleaning and disinfection procedures for PROPER components and suggest limited reuse of HEPA H14 corrugated filter cartridge, which can generally be discarded and replaced after one or more usages depending on the drop in its efficiency below a threshold. The filter cartridge can be reused until they become so clogged that they reduce airflow or become visibly soiled or damaged. Clogging is not expected in environments such as healthcare settings. The outside of the filter cartridge can have surface cleaning and decontamination while the rest of the PROPER unit is being serviced. Following the “Steps for cleaning and disinfecting PAPR components” in [5], the disinfection of PROPER components (with the exception of 3D printed components) is done using a clean, soft cloth dampened with warm water approximately 49 °C (120°F) containing a mild pH neutral (pH 6–8) detergent and using a mechanical wiping action. Any component exposed to moisture during the cleaning process needs to be carefully and thoroughly dried. Pay attention to never soak, dip, or immerse the motor/blower assembly or powerbank and related electronics in disinfectant.

The 3D printed components of PROPER can be disinfected using similar procedure as the rest of the PROPER components or by fully immersing the components in the solution for 60 min. The components are then removed from the solution and wiped with the same solution and thoroughly dried.

### 6.4 Tuning the PROPER

**Fig. 17:**
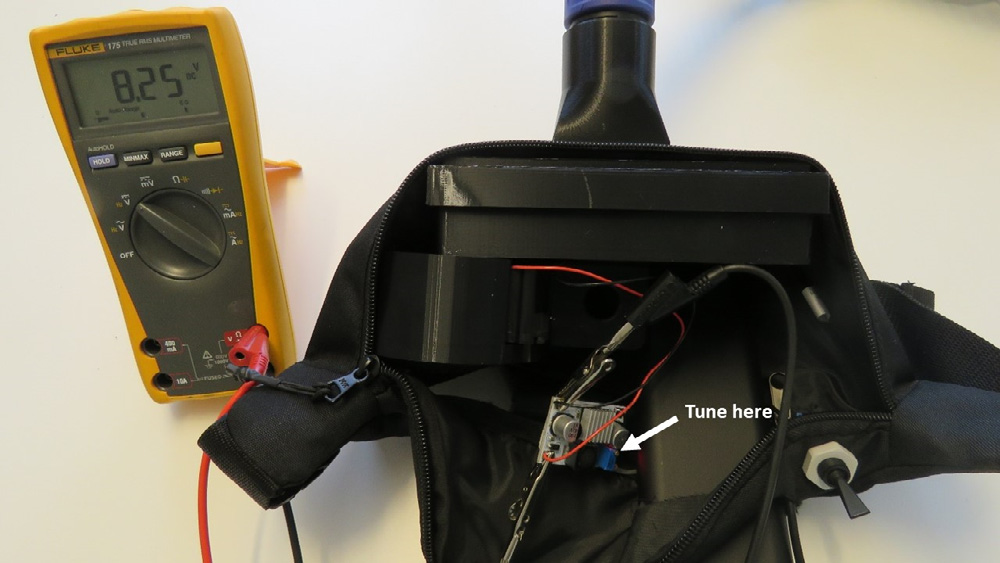
The air flow of the PROPER can be preset by tuning the tiny screw potentiometer to a desired input voltage to the centrifugal fan. The multimeter read 8.25 V during our operation.

The centrifugal fan of the PROPER is designed to provide a constant air flow at a specific static pressure. However, the input voltage supplied to the centrifugal fan can be varied in order to tune the unit to provide a required air flow anywhere between 6 and 8.8 CFM. The adjustable DC-DC step-up module serves this purpose. The module includes a tiny screw potentiometer that can be tuned with a small flat screwdriver until the desired output voltage is achieved. This voltage is then supplied to the centrifugal fan to obtain a customized air flow. With the current design, PROPER is set at a constant air flow by tuning the voltage prior to its operation. Controlling the air flow during operation can be achieved by adding a PWM motor driver module to adjust the voltage in real-time. Fig. 17 shows that we have tuned the adjustable DC-DC step-up module to 8.25 V. The reason being, at voltages greater than this, an increased air flow caused an additional pressure drop allowing a backflow of air to occur into the centrifugal fan. This triggered an uncomfortable noise and is also not good for the fan motor during longer operations.

## 7 Validation and characterization

Considering the NIOSH procedures for field testing respirators [30] and field-testing method for loose-fitting PAPRs equipped with HEPA filters [31] as reference, we performed the validation procedures to verify the compliance of PROPER with the required performance characteristics of air flow, cleanliness level, gas concentrations and overpressure. The validation tests we have run do not constitute certification, but it is a demonstration of the operability of the PROPER equipment. Upon completion of these tests, PROPER is eligible for certification according to each country’s policies. The procedures and their outcomes are described below in this section.

### 7.1 Air flow

**Fig. 18:**
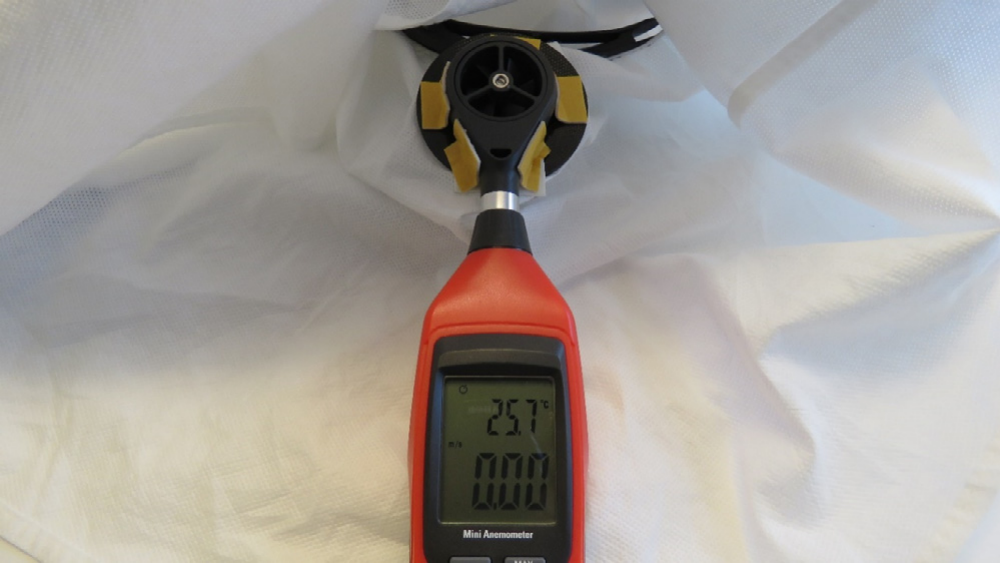
Experiment setup to determine the air flow inside the hood of PROPER during operation.

**Fig. 19:**
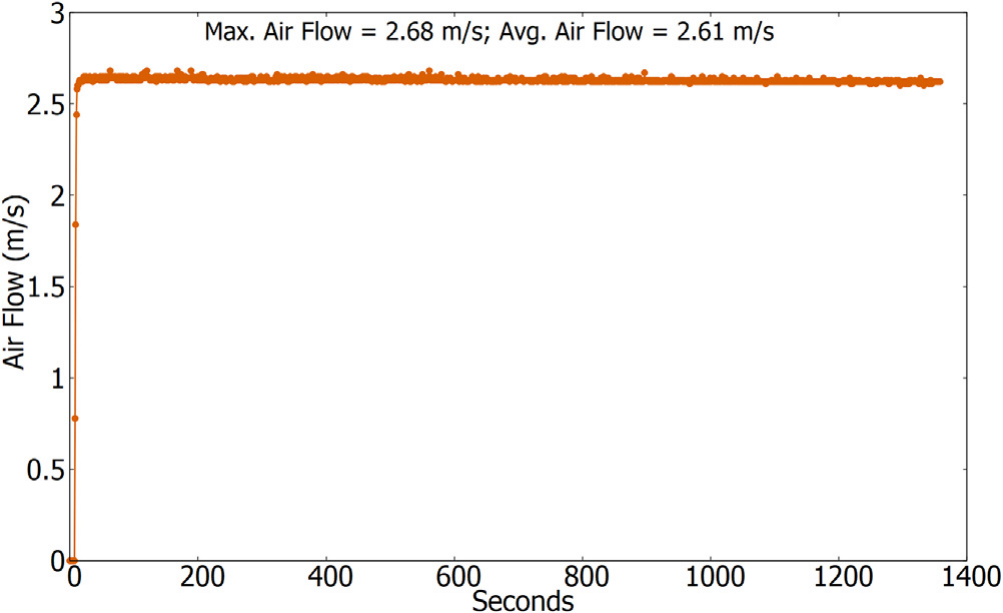
Air flow inside the hood of PROPER during 24 min of nominal operation. Maximum and average air flow are also shown.

To determine the air flow inside the hood of PROPER, we measured the air velocity at the inner side of the hood adaptor which is the entry point of air into the hood after travelling through the breathing tube. We used an anemometer (RS Pro RS-90 Mini Anemometer) with a range of 1.10–25.00 m/s at a resolution of 0.01 m/s and accuracy of ±(3% + 0.30 m/s). Fig. 18 shows the experimental setup for air flow measurement. The anemometer was directly attached to the inner side of the hood adaptor with the help of double-sided white foam tape and any disturbance from the ambient environment is cut off by folding the shroud of the hood fabric around the base of the anemometer and securing with a strip of masking tape to provide an airtight seal. Fig. 19 shows the air flow results during half an hour of nominal PROPER operation.

Air flow (in m^3^/s) can be calculated from the determined air velocity (in m/s) by multiplying the cross-sectional area (in m^2^) of the inner side of the hood adaptor as represented in equation (1).

(1) $$\mathrm{Air}\;flow=Air\;velocity\times (\pi \times {\mathrm{Radius}\;of\;hood\;adaptor}^{2})$$

For an average air velocity of 2.61 m/s and a radius of hood adaptor of 0.017 m,

$$\mathrm{Maximum}\;Air\;flow=2.61\times \left(3.14\times 0.017^{2}\right)=0.00236847\;m^{3}/s\approx 5.0185\;CFM$$

For the achieved maximum air velocity of 2.61 m/s, the pressure drop across the HEPA filter is computed from Fig. 2 to be 1.21 in.-H_2_O. However, the achieved air flow of 5.0185 CFM is slightly lower than the recommended minimum air flow of 6 CFM for a PAPR system. There has been additional pressure drop than we accounted for, that led to a lower air flow for the specific static pressure requirement induced by the prefilter, HEPA filter and tubing. This can be corrected by replacing the centrifugal fan with the one having a higher static pressure, typically over 2.5 in.-H_2_O or about 600 Pa.

### 7.2 ISO class cleanliness level

**Fig. 20:**
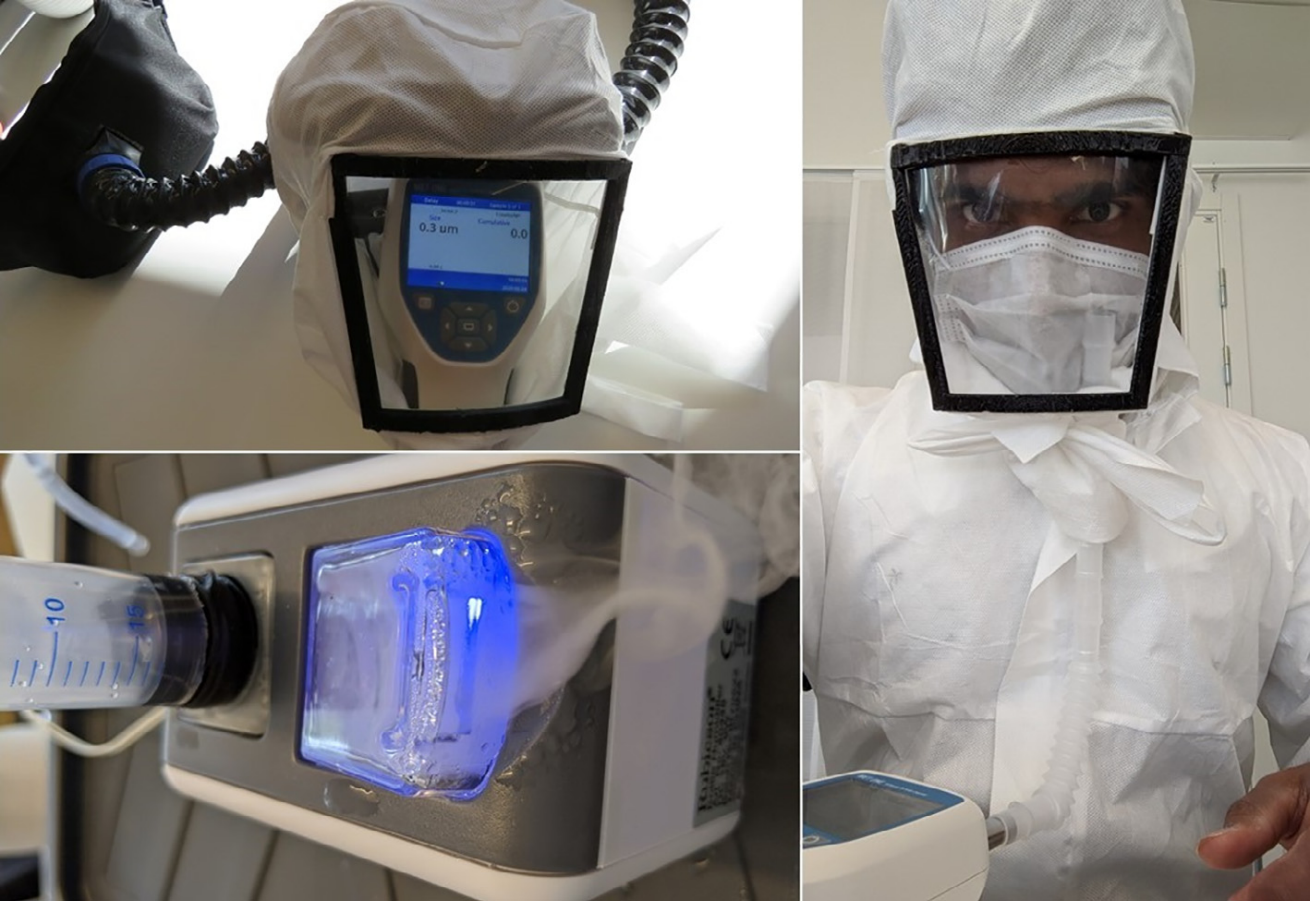
Experiment setup to determine the particle count (left top) inside the hood of PROPER during operation without operator, and (right) with operator. (bottom) Humidifier used to produce the aerosol environment.

To determine the cleanliness level inside the hood of PROPER, we measured the count of 0.3 μm size particles within the enclosed cleanroom environment inside the hood. We used a Handheld Airborne Particle Counter (MET ONE HHPC 6+) with an analysis range of 0.3–10 μm particle size. The throughput of the particle counter is 2.83 LPM. Fig. 20 shows the experimental setup for particle counting.

Since the area inside the hood, A is 0.2826 m^2^, the minimum number of locations to be sampled for particles is calculated [32] according ISO 14644-1 as expressed in equation (2),

(2) $$N_{L}=\sqrt{A}$$

The nearest whole number is 1, thus we sampled in one location. The head of the particle counter was placed directly inside the center of the hood and mixing of air from the ambient environment is cut off by folding the shroud of the hood fabric around the base of the particle counter and securing with a strip of masking tape to provide an airtight seal.

We measured the particle count with a target cleanliness class of ISO 6 with the maximum concentration limit (particles/m^3^ of air) of 102,000 particles equal to and larger than the considered 0.3 µm size [31]. The minimum single sample volume V_s_ per location in liters is expressed in equation (3).

(3) $$V_{s}=\left(\frac{20}{C_{n,m}}\right)\times 1000$$

where, *C_n_*_,_*_m_* is the class limit (number of particles per cubic meter) for the largest considered particle size (n = 0.3 µm) specified for the class limit (m = 6) = 102000 [32].

So, the minimum sample volume can be calculated to be 0.196 L, which at the sampling flow rate of 2.83 L per minute requires a sample time of 0.07 min. Since ISO 14644–1 places lower limits on minimum volume samples at each location as being at least 2 L and that sampling must be conducted for at least 1 min, the particle counting was performed for 1 min. To allow a smooth and constant air flow, a ten-minute delay was set in the particle counter when turning ON the PROPER. A control experiment was also performed counting the 0.3 μm size particles for 1 min in the ambient environment for comparison. Furthermore, an aerosol environment was prepared using a humidifier (Rubicson 40448) that generated a distribution of aerosols whose size has been quantified within the ranges of 0.3 μm. 0.5 μm, 1 μm, 2 μm, 5 μm and 10 μm. The particle concentration was counted inside the hood with the help of a sampling tube, see Fig. 20.

Table 4 shows the results of particle count inside the hood during PROPER operation (with and without operator) and in the ambient environment outside the hood (Sample 1 and 2). The results of operation of PROPER in the aerosol environment with different particle distribution (Sample 3) is also provided to demonstrate the efficiency in filtration.

**Table 4:** Comparison of particle count inside and outside the hood.

Counts/m^3^	Inside the hood (without operator)	Inside the hood (with operator)	Outside the hood
Sample 1 (2020/06/24) in normal laboratory ambient	0.3 μm: 39132.7	NA	0.3 μm: 4702473.5
Sample 2 (2020/08/24) in normal laboratory ambient	0.3 μm: 61484.1	0.3 μm: 71024.7	0.3 μm: 12,518,375
Sample 3 in laboratory with aerosols	0.3 μm: 52650.2	0.3 μm: 180,212	0.3 μm: 98,581,272
	0.5 μm: 4250.3	0.5 μm: 24,735	0.5 μm: 72,768,552
	1 μm: 706.7	1 μm: 6007.1	1 μm: 66,748,056
	2 μm: 706.7	2 μm: 2826.9	2 μm: 61,901,412
	5 μm: 353.4	5 μm: 706.7	5 μm: 31,882,332
	10 μm: 0.0	10 μm: 0.0	10 μm: 6,207,067
Limit for ISO 6 class	102,000		

The result for the area inside the hood produces 95% Upper Confidence Interval less than the ISO Class limit and therefore this area was certified as being, ISO Class 6. However, the particle counts measured in the ambient environment, under normal laboratory conditions, shows a result that is over two orders of magnitude higher than the area inside the hood. ISO 14644-1 doesn’t provide a maximum concentration limit above ISO Class 6 for 0.3 µm particle size. Hence, we deem that the measured particle count in the ambient environment is over ISO Class 6. With this procedure, we validated that the area inside the hood is more than 200 times cleaner than the ambient without the operator and more than 175 times with the operator. Similarly, within the aerosol environment, the interior of PROPER is more than 1800 times cleaner (0.3 μm) than the exterior without operator and more than 500 times (0.3 μm) with the operator. Also, the fractional capture efficiency T(0.3 μm) of the HEPA filter for particle size 0.3 μm and higher can be calculated [33] as expressed in Eq. (4). Table 5 shows the results.

(4) $$T\left(0.3\;\mu m\right)=1-\frac{c(0.3\;\mu m)}{c_{o}\left(0.3\;\mu m\right)}$$

where, *c_o_* = concentration in unpurified air (outside the hood) and *c* = particle concentration of purified air (inside the hood without the operator).

**Table 5:** Fractional capture efficiency of the HEPA filter used in PROPER for the particle size of 0.3 μm at the achieved air velocity of the system.

Sample	Fractional capture efficiency (%)
Sample 1 (2020/06/24) in normal laboratory ambient	99.17
Sample 2 (2020/08/24) in normal laboratory ambient	99.51
Sample 3 in laboratory with aerosols	99.95

After every use of PROPER, the equipment is dismantled, disinfected, and reassembled on a new garment and hood inside a clean environment. This prevents the contamination of PROPER components and ensures that the particle count is within the certified ISO class. However, since the measured particle count and the certified ISO class 6 is without an operator (due to practical difficulty of fitting the particle counter with the operator), we foresee that additional particles from operator’s hair and breathing may be introduced inside the hood. However, all these particles would come from the healthy operator and most of them would be removed with wipes during the cleaning phase. The purpose of the particle count validation is to demonstrate that the use of a fan with a filter, allows to reduce by two order of magnitude the income of particles above 0.3 µm size into the “wearable clean zone”.

### 7.3 Oxygen and carbon-dioxide concentration

**Fig. 21:**
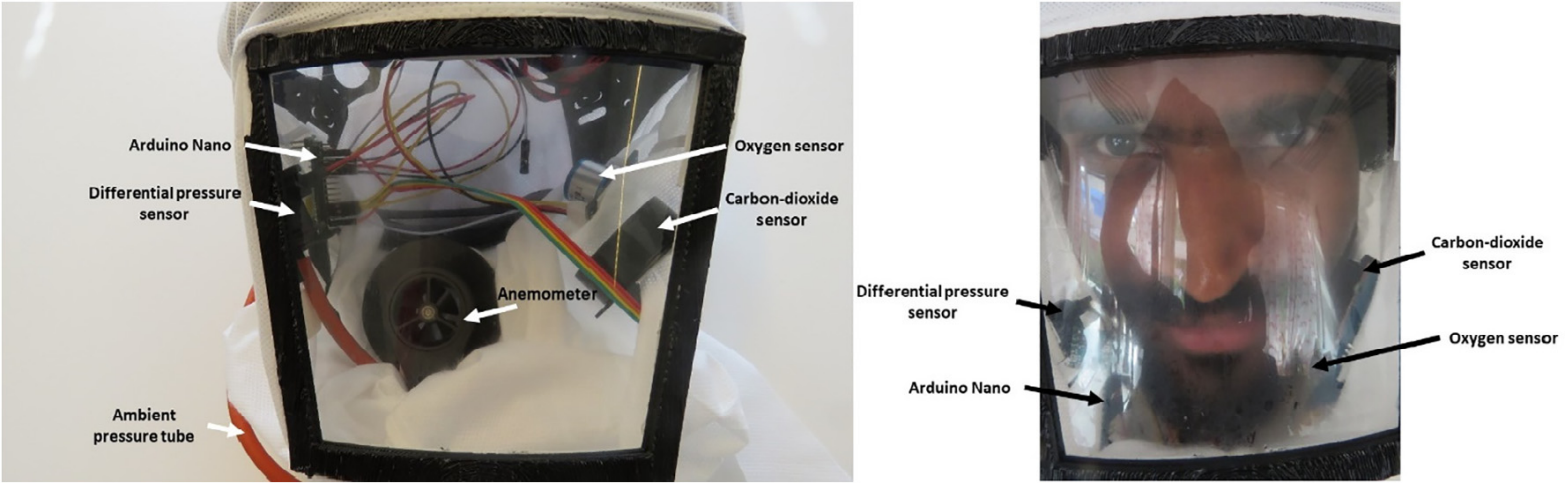
Experiment setup to determine the oxygen, carbon-dioxide gas concentrations and overpressure inside the hood of PROPER during an hour of continuous demonstration, (left) without the operator inside and (right) with the operator inside.

To verify that the exhaled gas is safely “leaked” out through the fabric and other tiny air gaps and to determine the buildup of gases during inhalation and exhalation inside the hood of PROPER, we measured the oxygen, O_2_, and carbon-dioxide, CO_2_, gas concentrations within the enclosed environment inside the hood as recommended by the CDC guidelines for testing PAPR [34]. This experiment was performed for an hour. We used an oxygen sensor (Grove, ME2-O2-Ф20) with a range of 0–25% for oxygen measurement. A carbon-dioxide sensor (Sensiron, SCD30) with a range of 0–40,000 ppm and a resolution of 1 ppm was used for carbon-dioxide measurement at the mouth as per the recommendations [34]. In addition, a differential pressure sensor (Sensiron, SDP2000-L) with a range of −100 Pa to 3500 Pa was used to sense the overpressure inside the hood. The reference pressure of the ambient environment was provided to the sensor through a long flexible tube protruding out through the open bottom of the hood. Fig. 21 shows the experimental setup for gas concentrations and pressure measurements. The gas sensors, differential pressure sensor, and an Arduino Nano were placed directly inside the hood around the head. For control experiment without donning the hood, the mixing of air from the ambient environment is cut off by folding the shroud of the hood fabric around the ambient pressure tube and the USB cable for Arduino Nano, and securing with a strip of masking tape to provide an airtight seal.

**Fig. 22:**
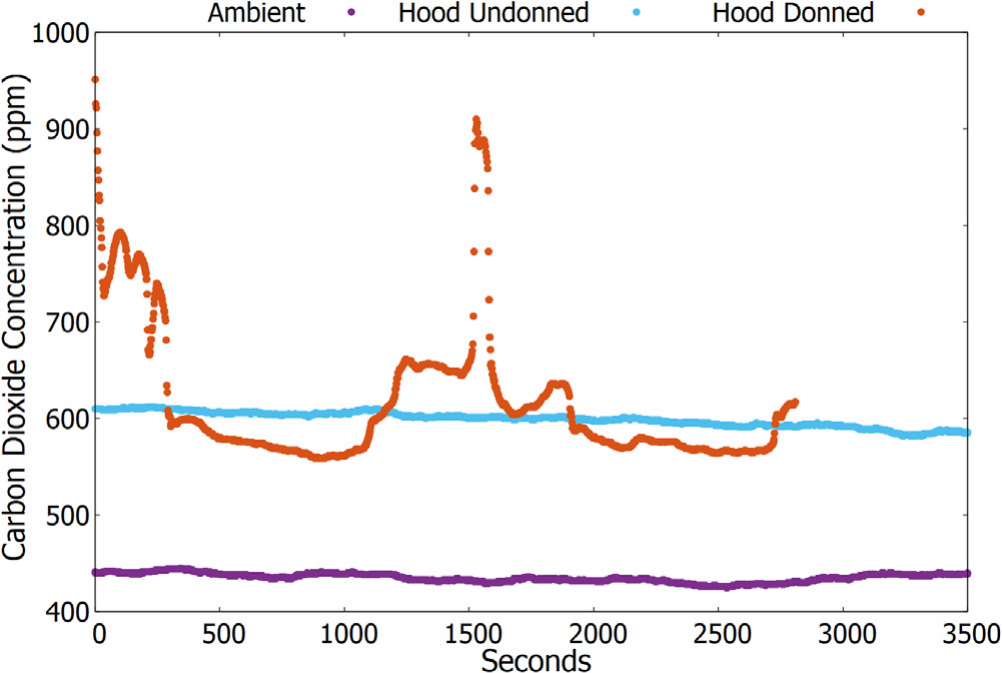
Comparison of gas concentrations in ambient environment, inside the hood during operation without an operator and after donning the PROPER. A sudden spike in the middle of the orange curve was due to blowing into the sensor simulating an excess carbon-dioxide production during heavy physical activity. As soon as this exercise is over, the carbon-dioxide concentration reduced back to around 550 ppm.

Fig. 22 show a comparison of the evolution over time of gas concentrations inside the hood during an hour of nominal PROPER operation, without an operator, and while it is donned. The carbon-dioxide concentration inside the hood spiked up until the PROPER was turned ON. The gas concentration gradually reduced as shown in Fig. 21 (orange curve), as a result of fresh purified air being pumped in the hood at a rate of about 5 CFM while maintaining an overpressure of 0 to 4.56 Pa with occasional buildup up to 9.12 Pa. The carbon-dioxide concentration stabilized around 570 ppm (within typical range of 400–1000 ppm in occupied indoor spaces with good air exchange [35]) around the same level as when PROPER was in operation without donning (cyan curve). The average ambient carbon-dioxide concentration outside the hood was 435.11 ppm. The oxygen concentration was always hovering around the nominal 21% (not shown). The average temperature inside the hood during the hour operation as 30.6 °C with an average relative humidity of 36.3%. These experiments show that the gas concentrations and the conditions inside the hood during an hour of continuous operation of PROPER is within the desired limits to be operated safely.

### 7.4 Power consumption and battery life

The PROPER unit consumes about 5 V, 1.6 A power that is supplied by a 15,600 mAh powerbank. A long duration experiment revealed that the lifetime of the battery is about 8–9 h. It takes about 9 h to fully recharge the battery through a micro-USB port (max. 2.1 A). We recommend having one or two additional powerbanks assigned for each PROPER unit to support its continuous operation.

## 8 Conclusion

PROPER is a portable clean room area, adapted for the use in clinical environments. PROPER’s simple and efficient open source design will be a new addition to the efforts to ensure safety and protection of HCPs against the SARS-CoV-2 virus. While the qualified PPE such as PAPRs are of limited availability and the regular masks are in shortage, PROPER would ensure easy and fast manufacturing with the help and support from the public and maker’s community. In the current pandemic environment, PROPER would be able to offer a better protection than an N95 mask, mainly because it is insensitive to seal fit and it shields the eyes as well. While the Emergency Use Authorization (EUA) remains in place, PROPER could offer a workable patch for some of the demand for respirators and is recommended for use during the peak shortage of a proper PPE for HCPs around US and globally, particularly in developing countries. PROPER can be reused, by cleaning and sterilizing most of its parts and substituting the garment and hood by a new unit.

## 9 Disclaimer

The designs and other information (the “Design”) made available in this article is at an early stage of development. Accordingly, specific results are not guaranteed, and the Design provided here is provided “AS IS” and without any express or implied warranties, representations or undertakings. As examples, but without limiting the foregoing, the University of Aberdeen, Luleå University of Technology, Instituto Andaluz de Ciencias de la Tierra (CSIC-UGR), Centro de Astrobiología (CSIC-INTA) and their employees and students do not give any warranty or guarantee that the Design is of merchantable or satisfactory quality, is fit for any particular purpose, complies with any sample or description including the requirements for medical device registration, or are viable, uncontaminated, safe or non-toxic, accurate, up to date or complete. The University of Aberdeen, Luleå University of Technology, Instituto Andaluz de Ciencias de la Tierra (CSIC-UGR), Centro de Astrobiología (CSIC-INTA) and the authors have not performed any searches or investigations into the existence of any third-party rights that may affect the Design. Anyone may use the Design entirely at their own risk, and the University of Aberdeen, Luleå University of Technology, Instituto Andaluz de Ciencias de la Tierra (CSIC-UGR), Centro de Astrobiología (CSIC-INTA) and/or the authors are not liable for such use of the Design, including without limitation any direct or indirect losses. Any users of the design should appropriately attribute the author.

## 10 Human **and animal rights**

This work involved testing of the equipment on a human subject to validate the oxygen and carbon-dioxide gas concentrations. The test was performed on one of the authors and an informed consent was obtained for experimentation. We ensure that the work described has been carried out in accordance with The Code of Ethics of the World Medical Association (Declaration of Helsinki) for experiments involving humans; Uniform Requirements for manuscripts submitted to Biomedical journals.

## Declaration of Competing Interest

The authors declare that they have no known competing financial interests or personal relationships that could have appeared to influence the work reported in this paper.
